# An SPFH Protein Couples Membrane Stress to Differentiation in *Bacillus subtilis*

**DOI:** 10.1111/1462-2920.70467

**Published:** 2026-10-27

**Authors:** Sarah S. M. Baur, Urška Repnik, Tobias Busche, Louisa Rau, Alisa Mondry, Marc Bramkamp

**Affiliations:** ^1^ Christian‐Albrechts‐Universität zu Kiel, Mathematisch‐Naturwissenschaftliche Fakultät Institut für Allgemeine Mikrobiologie Kiel Germany; ^2^ Christian‐Albrechts‐Universität zu Kiel, Mathematisch‐Naturwissenschaftliche Fakultät Zentrale Mikroskopie Kiel Germany; ^3^ Bielefeld University Medical School OWL & Center for Biotechnology (CeBiTec) Bielefeld Germany; ^4^ MRC Laboratory of Molecular Biology Cambridge UK

**Keywords:** biofilm, BslA, flotillin, membrane, motility, SPFH, starvation

## Abstract

*Bacillus subtilis* adapts to fluctuating environmental stress, such as membrane perturbation or alkaline conditions, using membrane‐associated regulatory complexes. Here, we rename the previously termed *pspA‐ydjGHI* operon to *pspA‐samGHI* (for starvation and motility) to reflect its functional roles. The membrane proteins SamG and SamH recruit SamI, a cytosolic SPFH (Stomatin/Prohibitin/Flotillin/HflK‐C) protein that coordinates the recruitment of PspA, an ESCRT‐III homologue from a protein family involved in membrane repair and remodelling. Here we show that SamGHI impacts developmental processes, allowing proper motility, secretion, and colony biofilm formation in the laboratory *B. subtilis* strain WT168. Loss of SamI (Δ*samI*/Δ*ydjI*) leads to unbalanced SamH activity, resulting in global transcriptional changes reminiscent of starvation situations. This, in turn, reduces secretion of the matrix protein BslA, preventing colony biofilm formation. Deletion of *samH* in combination with Δ*samI* restores colony morphology, while Δ*pspA* mutants form normal colonies, indicating that PspA is dispensable for the developmental phenotype. Our findings reveal that, beyond its role in maintaining membrane integrity, SamGHI influences the cellular physiological state and thereby impacts multicellular development.

## Graphical abstract

The SPFH protein SamI and the integral membrane proteins SamG and SamH form the SamIGH complex, linking envelope stress signalling to developmental programs in *Bacillus subtilis* . Loss of SamI causes chronic envelope stress signalling and impairs biofilm development, resulting in reduced structural complexity. These findings identify SamIGH as a functional link between membrane homeostasis and bacterial differentiation.

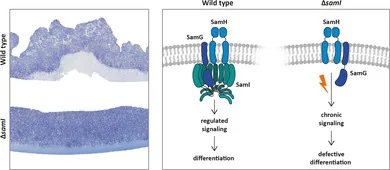

## 1 Introduction

*Bacillus subtilis* inhabits diverse environments, ranging from soil niches such as the plant rhizosphere to the gastrointestinal tract of animals (Earl et al. 2008; Stülke et al. 2023; Akinsemolu et al. 2024). These habitats are characterized by fluctuating conditions that require rapid physiological adaptation. In response to environmental cues, *B. subtilis* differentiates into distinct phenotypes that allow it to cope with, escape, or endure stress (López and Kolter 2010; Qin et al. 2022). One common lifestyle adopted in nature is biofilm formation, in which cells are embedded in a matrix composed of proteinaceous and carbohydrate‐rich fibres that protect the biofilm community from external stresses (Vlamakis et al. 2013; Mielich‐Süss and López 2015; Arnaouteli et al. 2021). Biofilm development is controlled by a complex regulatory network with Spo0A as the central master regulator (Branda et al. 2001; Hamon and Lazazzera 2001).

Activation of membrane‐associated sensor kinases initiates a phosphorelay that modulates the phosphorylation state of Spo0A. Importantly, biofilm formation is not simply a consequence of Spo0A phosphorylation; rather, it is associated with the accumulation of an optimal, intermediate level of Spo0A~P, which relieves repression mediated by the master regulators SinR and AbrB and thereby promotes the expression of biofilm‐associated genes.

The sensor kinases KinC and KinD play prominent roles in biofilm formation. Constitutively active KinC generates intermediate levels of Spo0A~P, which are sufficient to induce expression of *sinI* (Chai et al. 2008; Chen et al. 2022). SinI antagonizes the biofilm repressor SinR by binding to and inhibiting its DNA‐binding activity, thereby relieving SinR‐mediated repression of *slrR* and other biofilm‐associated genes (Bai et al. 1993; Chai et al. 2008; Chen et al. 2023). SlrR can subsequently sequester SinR, further reinforcing the transition toward the biofilm state (Chai et al. 2010). KinD likewise promotes low‐level Spo0A phosphorylation and has been proposed to function as a checkpoint linking sporulation and biofilm development (Aguilar et al. 2010). In parallel, Spo0A~P represses *abrB* expression, resulting in reduced AbrB levels toward the end of exponential growth (Kobir et al. 2014). Collectively, these regulatory pathways alleviate SinR‐ and AbrB‐mediated repression and establish a transcriptional state permissive for biofilm development. Inactivation of the repressors AbrB and SinR induces the expression of the matrix gene clusters *epsA–O*, *tapA–sipW–tasA*, and *bslA* (Hamon et al. 2004; Kearns et al. 2005; Verhamme et al. 2009; Kovács and Kuipers 2011). The resulting extracellular polysaccharides and TasA amyloid fibres constitute the core structural components of the biofilm matrix, while BslA forms a hydrophobic surface layer that protects the biofilm (Branda et al. 2001, 2006; Kobayashi and Iwano 2012).

In addition to Spo0A‐dependent regulation, alternative sigma factors further fine‐tune cellular differentiation. *B. subtilis* encodes 18 alternative sigma factors that modulate transcriptional responses to diverse stresses (reviewed in (Collins et al. 2023)). Among them, σ^W^ is activated by membrane stress such as phage infection, antibiotic exposure, and alkaline or high‐salt conditions (Petersohn et al. 2001; Wiegert et al. 2001; Wenzel et al. 2012). σ^W^ regulates 37 operons, including three that encode SPFH (Stomatin/Prohibitin/Flotillin/HflK‐C) family proteins (Wiegert et al. 2001; Cao et al. 2002).

SPFH proteins are widespread in all domains of life and are defined by a conserved SPFH domain (Langhorst et al. 2005; Rivera‐Milla et al. 2006; Hinderhofer et al. 2009; Bramkamp and López 2015). Although their precise functions remain incompletely understood, they are broadly associated with membrane organization and homeostasis (Tatsuta et al. 2005; Browman et al. 2007; Boehm et al. 2009). The SPFH domain promotes self‐oligomerization (Tavernarakis et al. 1999; Boehm et al. 2009), leading to the formation of ring‐like megacomplexes that associate with cellular membranes (Ma et al. 2022; Tan et al. 2024).

Two SPFH‐domain proteins in *B. subtilis* , FloA and FloT, have been studied extensively. Both are membrane‐associated proteins, and deletion of *floA* or *floT* results in distinct alterations in membrane organization and cellular physiology (Bach and Bramkamp 2013; Bach and Bramkamp 2015; Bramkamp and López 2015; Zielińska et al. 2020; Álvarez‐Mena et al. 2025). A third SPFH protein, SamI (formerly YdjI), is encoded at the terminal position of the *pspA*–*samGHI* operon. In this study, we refer to YdjI as SamI (for starvation and motility) based on the observed phenotypes upon deletion and accordingly rename YdjG and YdjH to SamG and SamH, respectively. SamI is encoded at the terminal position of the *pspA*–*samGHI* operon. It differs structurally from better characterized SPFH domain proteins FloA and FloT, containing a C‐terminal zinc finger motif, lacking an intrinsic membrane anchor, and instead carrying a low‐complexity region between the SPFH domain and the zinc finger motif (Scholz et al. 2021). However, similar to deletion of *floA* and *floT*, deletion of *samI* decreases membrane fluidity in vivo (Scholz et al. 2021). Membrane association of SamI is mediated by the integral membrane proteins SamG and SamH. SamG further encodes for a zinc finger motif, located in the cytoplasm and SamH encodes for an extracellular TPM domain (TLP18.3, Pbs32, and MOLO‐1) (Scholz et al. 2021), a domain that is associated with phosphatase activity (Wu et al. 2011; Sinha et al. 2015; Pellizza et al. 2016). SamI is additionally required for the membrane recruitment and foci formation of PspA, an ESCRT‐III family protein with roles in membrane maintenance and remodelling (McCullough et al. 2015; Thurotte et al. 2017). ESCRT‐III proteins form large oligomers capable of binding and deforming membranes, supporting membrane fusion and fission, and the stabilization of stressed membrane regions (Heidrich et al. 2017; Thurotte et al. 2017; Liu et al. 2021; Junglas et al. 2021; Junglas, Kartte, et al. 2025; Hudina et al. 2025; Junglas, Hudina, et al. 2025; Melnikov et al. 2025). Together, the PspA–SamGHI complex is thought to function in membrane stress sensing or response (Scholz et al. 2021; Bramkamp and Scheffers 2023).

Although SamI has been implicated in membrane organization (Scholz et al. 2021), its physiological role in *B. subtilis* remains largely unexplored. Here, we investigate SamI's function during two differentiation programs, colony biofilm morphology and cell motility. Importantly, deletion of *samI* results in a strong colony phenotype in the domesticated laboratory strain WT168, making this strain background ideal to dissect the function of SamI. In contrast, no apparent biofilm phenotype is observed upon deletion of *samI* in the undomesticated strain 3610, which forms substantially more robust biofilms. Thus, the phenotype of Δ*samI* is dependent on the strain background. Our data suggest that the membrane‐associated SamGHI complex is involved in maintaining proper cellular function leading to metabolic changes upon loss of SamI, which result in incomplete differentiation. Importantly, deletion of the entire operon suppresses the observed phenotype, indicating that SamI is required to regulate the function of SamH spatially or temporally.

## 2 Materials and Methods

### 2.1 Strains and Media

*B. subtilis* strains derived from the WT168 (lab strain originating from Groningen University) or the NCIB 3610 (DSM 10, ATCC 6051) background are listed in Table S1 and were grown in LB medium (5 g/L yeast extract, 5 g/L NaCl, 10 g/L tryptone, adapted from (Lennox 1955)) or in MD medium (10.7 g/L K_2_HPO_4_, 6 g/L KH_2_PO_4_, 1 g/L Na_3_C_6_H_5_O_7_ × 5H_2_O, 2% glucose, 50 μg/mL L‐tryptophan, 11 μg/mL ferric ammonium, 2.5 mg/mL L‐aspartate, 3 mM MgSO_4_, adapted from (Anagnostopoulos and Spizizen 1961)) supplemented with 1 μL/mL casamino acids at 37°C under shaking conditions. *Escherichia coli* strains (Table S2) were propagated in lysogeny broth (5 g/L yeast extract, 10 g/L NaCl, 10 g/L tryptone, adapted from (Bertani 1951)) at 37°C under shaking conditions. Growth media were supplemented with kanamycin (5 μg/mL), erythromycin (1 μg/mL), tetracycline (10 mg/mL), spectinomycin (100 μg/mL), chloramphenicol (5 μg/mL), carbenicillin (100 μg/mL), IPTG (Isopropyl β‐d‐1‐thiogalactopyranoside, 1 mM), or benzyl alcohol (0.05%–0.2%) when necessary. Plates were solidified with 1.5% agar unless stated otherwise.

### 2.2 Strain Construction

All plasmids generated in this study are listed in Table S3 and oligonucleotides used in this study are listed in Table S4.

Most single gene deletion strains derived from the *B. subtilis* gene deletion library (Koo et al. 2017). These strains were generated by transforming genomic DNA isolated from library strains into the WT168 and NCIB 3610 background using their natural competence. Generation of all knock‐out strains of the *pspA‐samGHI* operon was verified with primer SB001 and SB052. Flotillin mutants Δ*floT* (Donovan and Bramkamp 2009), Δ*floA* (Bach and Bramkamp 2013), and the full operon knock‐out Δ*pspA‐samGHI* were transformed in the here used WT168 generating SB002 (Δ*floT*), SB071 (Δ*floA*), and SBB007 (Δ*pspA‐samGHI*), respectively.

To transform *B. subtilis* , strains single colonies were inoculated in 10 mL MD medium supplemented with 50 μL casamino acids (20%) and grown to an OD_600_ of 1.0–1.5 (adapted from (Anagnostopoulos and Spizizen 1961)). 10–15 mL MD medium lacking casamino acids were added and after 1 h of incubation 800 μL of the cell culture were transferred and the donor DNA was added to the culture. After 20 min of incubation, 25 μL of casamino acids were added and strains were further incubation for 60–80 min before plating on LB plates with respective antibiotics. Transformation plates were incubated overnight (ON) at 37°C. If necessary integration into the *amyE* locus was verified with 0.3%–1% starch plates testing for amylases sensitivity and intact *ldh* gene was verified with primer SB137 and SB138 (Dierksheide and Li 2024).

For removal of the antibiotic cassette, strains generated with the gene deletion library were transformed with the plasmid pDR244 following the description of (Koo et al. 2017). Loss of the antibiotic cassette was confirmed by sequencing.

To extract genomic DNA a fresh colony from the respective strain was inoculated in 2.5 mL of LB medium and grown until the culture lost its motility for 3–4 h (adapted from (Ward and Zahler 1973)). Cell pellets were resuspended in 1 mL SCC (0.15 M NaCl, 0.01 M Na_3_C_6_H_5_O_7_, pH 7.0) supplemented with 0.2 mg/mL lysozyme and incubated for 15 min at 37°C until cell suspension was lysed. 1 mL of 4 M NaCl was added to induce osmolysis. The lysed cell suspension was filtered (0.45 μm) and stored, if necessary, at −20°C.

The plasmids used in this study (Table S3) were generated with Gibson Assembly (Gibson et al. 2009) with the NEBuilder HiFi DNA Assembly Master Mix (E2621). Fragments for Gibson Assembly were amplified using Phusion High‐Fidelity DNA Polymerase (M0350) or Q5 High‐Fidelity DNA Polymerase (M0491). Plasmids based on pKill were verified with primers SB118 and SB119 and plasmids based on pDR111 were verified with SB035 and SB036.

For double and triple deletions of the *pspA‐samGHI* operon genes the primers SB159 to SB170 were used. gDNA from SBB001 and SBB070 and plasmids pKill‐*samI‐halo‐spec* (SBE023) and pKill‐*spec‐halo‐samI* (SBE024) were used as template. Primers SB165 to SB170 were used for pKill‐samHI, primers SB159 to SB164 were used for pKill‐*samGH*, and primers SB160 to SB166 were used for pKill‐*samGHI*. The resulting plasmids were transformed into competent *E. coli* DH5α and plasmids were isolated, verified, and sequenced from the resulting colonies. Upon verification of the correct sequence, plasmids were linearized and transformed into SBB001, generating SBB138 (Δ*samHI*), SBB159 (Δ*samGH*), and SBB160 (Δ*samGHI*).

For complementation with *samI* under an IPTG inducible promoter in the *amyE* locus the primers SB027 to SB030 were used and the plasmid pDRyuaB2 (Kovács and Kuipers 2011) and genomic DNA of SBB001 was used as a template. The resulting plasmid pDR111‐*samI* was transformed into competent *E. coli* DH5α and from the resulting, verified colonies plasmids were isolated and sequenced. Upon verification of the correct sequence, plasmids were linearized and transformed into SBB003, generating SBB060 (Δ*samI* P_hs_‐*samI*). Transformation of genomic DNA from SBB070 into SBB060 resulted in SBB067 (Δ*samG* Δ*samI* P_hs_‐*samI*).

SamH was complemented via an *amyE* integration of *samH* under an IPTG inducible promoter. Primers SB028 and SB257 to SB259 were used and the plasmid pDRyuaB2 (Kovács and Kuipers 2011) and genomic DNA of WT168 was used as a template. The resulting plasmids pDR111‐*samH* was transformed into competent *E. coli* DH5α and from the resulting colonies plasmids were isolated and sequenced. Upon verification of the correct sequence, plasmids were linearized and transformed into SBB003, generating SBB193 (Δ*samI* P_hs_‐*samH*).

To generate the fragments for BslA‐His expression (*bslA::bslA‐6xHis‐cat*) primers SB037 to SB038, SB104 to SB106, SB111, SB114 to SB115, SB118, and SB119 were used. Fragments generated with primers SB037 to SB038, SB104 to SB106, SB111, SB114 to SB115 were used for Gibson assembly and the resulting construct was amplified again with SB118 and SB119. This fragment was verified by sequencing and directly transformed into *B. subtilis* SBB041 generating SBB131. DNA was isolated from SBB131 and retransformed into SBB001 and SBB003 generating SBB127 (BslA‐His) and SBB129 (Δ*samI* BslA‐His), respectively.

For generation of a *divIVA‐gfp* fusion DNA was isolated from 1803 (Edwards et al. 2000) and transformed into SBB001 and SBB003 generating SBB133 (DivIVA‐GFP) and SBB134 (Δ*samI* DivIVA‐GFP).

Generation of P_*yxl*_ *‐gfp* was achieved by transforming pSG1729 (Lewis and Marston 1999) into SBB001 and SBB003, generating SBB102 (P_*yxl*_ *‐gfp*) and SBB245 (Δ*samI*, P_*yxl*_ *‐gfp*).

For the *fliM*‐GFP fusions, gDNA of a Δ*fliM* from the gene deletion library was transformed into SBB001 and SBB094, generating SBB149 (Δ*fliM*) and SBB150 (Δ*samI* Δ*fliM*). These strains were further transformed with gDNA isolated from the strain DS8521 (Guttenplan et al. 2013) and generating SBB151 (Δ*fliM* P_fla/che_ FliM‐GFP) and SBB152 (Δ*samI* Δ*fliM* P_fla/che_ FliM‐GFP), respectively.

For generation of msf*gfp*‐*minD* fusion the plasmid of pHF2 (Feddersen et al. 2021) was transformed into SBB001 and SBB003 generating SBB246 (msf*gfp*‐*minD*) and SBB247 (Δ*samI*, msf*gfp*‐*minD*).

### 2.3 Growth Curves

Overnight cultures were inoculated from a fresh plate in minimal (MD) or full (LB) medium with respective antibiotics. Cultures were diluted with their respective medium to an OD_600_ of 0.1 in the next morning and growth was monitored over the next 8 h. An additional timepoint was measured after 24 h.

### 2.4 Biofilm Assay

Unless stated otherwise, biofilm assays were always performed with LBGM medium (LB medium supplemented with 0.1 mM MnSO_4_ and 1% (v/v) glycerol (Shemesh and Chai 2013)). The plates for biofilm assays were poured in the morning before inoculation, consisting of 25 mL LBGM medium or MSgg medium (1.925 mM KH_2_PO_4_ (pH 7.0), 3.075 mM K_2_HPO_4_ (pH 7.0), 100 mM MOPS (pH 7.0), 0.5% glutamate, 2 mM MgCl_2_, 700 μM CaCl_2_, 50 μM MnCl_2_, 1 μM ZnCl_2_, 2 μM thiamine, 50 μg/mL phenylalanine, 50 μg/mL tryptophan, and 0.5% glycerol) (Branda et al. 2001). 50 μM FeCl_3_ was added to MSgg medium on the day of the experiment. Cultures were grown by picking a fresh colony and inoculating it into LB medium. After reaching an OD_600_ of 0.4 to 0.8, cultures were diluted to an OD_600_ of 0.1, IPTG (1 mM) was added when necessary, and grown until they reached an OD_600_ of 0.8 to 1.0 again. The OD_600_ was normalized to 0.8, and 1.5 μL were inoculated onto LBGM or MSgg plates or into 1.5 mL LBGM medium in a 24‐well plate. Biofilm assays were always performed in technical triplicates and at least three biological replicates. Agar and medium were supplemented with 0.05%–2% benzyl alcohol or 1 mM IPTG when indicated. The biofilm plates were incubated at 30°C for 48 h (LBGM based medium) or 72 h (MSgg based medium), and the pellicle development was observed at room temperature for 1 week. Growth was documented with a ChemiDoc MD (Bio‐Rad). For further isolation of cells, RNA, or protein, biofilms were harvested after 48 h.

Contact angles of biofilms were determined after 48 h (for LBGM) or 72 h (for MSgg) of growth by pipetting a 5 μL drop of deionized water, stained with Coomassie Brilliant Blue for increased contrast (Hobley et al. 2013). The drop was left for 5 min to equilibrate before an image was taken from the side with a camera of a Pixel 7a. Contact angles were analysed with the ImageJ plugin Contact Angle, and average contact angles were always determined for at least three biological replicates consisting of three technical replicates each.

### 2.5 Swimming Assays

Motility assays were performed on LB plates with 0.5% agar. Plates were poured the day before, dried for 30 min and stored at room temperature until the next day.

Overnight cultures were inoculated from a fresh plate into LB medium with respective antibiotics if necessary. The cultures were diluted 1:100 the next morning and grown until an OD_600_ of 0.3–0.8. Cultures were diluted again to an OD_600_ of 0.1 and, after reaching an OD_600_ of 0.4–0.7, cultures were concentrated to an OD_600_ of 10, and 5 μL of the cultures were inoculated into the center of the LB plates. Plates were dried for 10 min and placed at 37°C. Plates were kept in a humidity chamber, an airtight sealed box with a reservoir of water in it. Development was observed over a time course of 0 h to 20 h and documented with a ChemiDoc MD (Bio‐Rad).

### 2.6 Flow Cytometry

To investigate expression levels of fluorescent reporters a protocol was adjusted from (Vlamakis et al. 2008). Biofilms from each of the three biological replicates were harvested and resuspended in 500 μL PBS. Biofilms were disrupted by passing through a 23 g1 needle for 10–15 times and fixed with 4% paraformaldehyde for 7 min at room temperature. After fixation samples were washed with PBS, resuspended in 500 μL GTE buffer (50 mM glucose, 10 mM EDTA at pH 8, 20 mM Tris–HCl at pH 8) and stored at 4°C. Before flow cytometry samples were homogenized with sonication for 2–4 × 20 impulses (Sonifier 250 (Branson), duty cycle 20%, output control 2) with cooling intermissions on ice between cycles. Disruption of cell clusters was confirmed by microscopy. Samples were diluted in PBS and analysed with a CytoFLEX S (Beckman Coulter) equipped with a 488 nm laser. The CytoFLEX S was operated using the CytExpert software 2.6.0.105. Flow speed was adjusted to achieve between 1.000 and 10.000 events/s. For each replicate 100.000 events were detected.

### 2.7 Fluorescence Microscopy

For determination of FliM‐GFP localization, cells were picked from the center and periphery of the biofilm and resuspended in 5 μL PBS. 2 μL of cell suspension were added on a 1% agarose pad in PBS and imaged at the Axio Zeiss Imager M1 fluorescence microscope (EC Plan‐Neofluor 100×/1.30 Oil Ph3 objective) equipped with an AxioCam HRm camera. GFP was excited at 470 nm for 500 ms and fluorescence emission was captured at 509 nm. Digital images were acquired with AxioVision Release 4.6.3 and analysed and edited with Fiji ImageJ.

### 2.8 Electron Microscopy

Fixative was poured onto a biofilm growing on LBGM agar. For ultrastructural analysis by TEM or by SEM, samples were fixed with 1% glutaraldehyde in 200 mM HEPES (pH 7.4) for several days. After 10 min of initial fixation, biofilms were cut into narrow strips to improve fixation efficiency.

For epoxy resin embedding, samples were postfixed with 1% osmium tetroxide prepared in 1.5% potassium ferricyanide for 1.5 h on ice, stained with 2% uranyl acetate for 2 h and dehydrated using a graded ethanol series (50%–70%–80%–90%–96%–100%) and then acetone, all steps at room temperature for a minimum of 15 min per step. Samples were progressively infiltrated with epoxy resin over two days before resin polymerization. Ultrathin 80 nm sections were cut using a Leica UC7 ultramicrotome and a Diatome ultra diamond knife and were contrasted with saturated aqueous uranyl acetate solution and Reynolds' lead citrate. Grids were imaged with a Tecnai G2 Spirit BioTWIN transmission electron microscope (FEI, now Thermo Fisher Scientific), equipped with a LaB6 cathode and operated at 80 kV using the TEM User Interface software (v. 4.2 build 6173). Images were acquired with a CCD side‐entry MegaView III G2 camera using iTEM software (v. 5.0) (all Olympus Soft Imaging Solutions, now EMSIS). To prepare 1 μm semi‐thin epoxy resin sections, few alterations were made to the above protocol. Specifically, 0.015% ruthenium red and 50 mM L‐Lysine were added to the fixative for the initial 30 min fixation, and then fixation was continued in the fixative supplemented with only 0.015% ruthenium red. Osmium tetroxide postfixation was also carried out in the presence of 0.075% ruthenium red. Sections were mounted on microscope slides and stained with Richardson's solution (alkaline solution of azure II (50%) and methylene blue (50%)) (Richardson et al. 1960). Bright‐field images were acquired using a Zeiss Primostar microscope equipped with a Plan‐Achromat 40× objective (0.65 N.A.) and an Axiocam 105 colour camera, which was operated with a Zen Blue (v. 3.2.) software.

For SEM analysis, samples were first dehydrated with a graded ethanol series and then subjected to critical point drying using a CPD 030 (Bal‐tec). Dried specimens were mounted on adhesive carbon tape and sputter coated with a 3 nm platinum layer using a Safematic CCU 010 HV compact coating unit. Samples were imaged with a Zeiss Sigma 300 VP SEM operated at 1 kV accelerating voltage and using a secondary electron detector.

### 2.9 RNA Isolation

Colony biofilms were transferred into a tube and submerged in 800 μL DNA/RNA Shield supplemented with 10 mg/mL lysozyme. To homogenize, biofilms were first passed through a 23 g1 needle 10–15 times and then sonicated for 12 bursts (duty cycle 20%, output control 2). A 30 min incubation at 37°C under shaking conditions and subsequent sonification for 4 × 20 bursts lysed the cells, and debris were pelleted by centrifugation at max. speed for 2 min. To isolate total RNA, the protocol of the *Quick*‐RNA Miniprep Plus Kit (Zymo Research) was followed. Concentration of isolated RNA was measured with a NanoDrop and integrity was confirmed in a denaturing agarose gel (Aranda et al. 2012).

### 2.10 RT‐qPCR

Reverse transcription qPCR was performed using the Luna Universal One‐Step RT‐qPCR Kit (NEB E3005) following manufacturers description. 100 ng of RNA were used as template and oligonucleotides used are listed in Table S4 (primers SB080 to SB103, SB116 and SB117). Amplification was carried out with AriaMx Real‐Time PCR System (Agilent Technologies). *divIVA* amplification was used as reference and the fold change in expression of *spo0A*, *abrB*, *lip*, *degQ*, *sinR*, *bslA*, *epsA*, *tasA*, and *samI* was determined by $2^{-\Delta \Delta \mathrm{Cq}}$ for three independent replicates with three technical replicates each. Significances were calculated using one‐sample *t*‐test with *p*‐value ≤ 0.05 and results were depicted within the graphs (ns—non‐significant, *p ≤ 0.05, and **p ≤ 0.005).

### 2.11 RNA Preparation

Sample purity and integrity was verified with Xpose and Agilent Bioanalyzer and DNA contaminated samples were treated with DNase Kit (Qiagen) and rechecked. RNA with an RNA Integrity Number (RIN) > 9 and an rRNA Ratio [23S/16S] > 1.5 and free of DNA was used for sequencing library preparation and sequencing.

### 2.12 Whole Transcriptome Sequencing

High‐quality total RNA aliquots were rRNA‐depleted using Pan‐Bacteria riboPOOL (siTOOLs BIOTECH). Successful rRNA depletion was confirmed using Agilent RNA Pico 6000 kit on Agilent 2100 Bioanalyzer (Agilent Technologies). The TruSeq Stranded mRNA Library Prep Kit from Illumina, starting with fragment, prime, and finish was used to prepare the cDNA libraries. The cDNA libraries were then sequenced in paired‐end mode on an Illumina NextSeq2000 system with 50 nt read length and a minimum of 5 million cluster per sample.

The resulting sequence reads were trimmed with Trimmomatic v0.33 (Bolger et al. 2014) to a minimal length of 35 base pairs and subsequently mapped onto the *Bacillus subtilis* subsp. subtilis str. 168 genome reference sequences (NC_000964.3) using Bowtie 2 (Langmead and Salzberg 2012). The ReadXplorer software version 2.0 (Hilker et al. 2016) and the integrated DESeq2 algorithm (Love et al. 2014) were used for the visualization of the mapped reads and the differential gene expression analysis, respectively. Differentially expressed targets were filtered with a log_2_ fold change (m‐value, log_2_FC) ≥ |1| and an adjusted *p*‐value ≤ 0.01 and the −log_10_P was plotted against the log_2_FC.

### 2.13 Sample Preparation for Immunoblotting

#### 2.13.1 Isolation of Biofilm Matrix

To isolate matrix proteins from biofilms the following protocol was adjusted (Branda et al. 2006; Arnaouteli et al. 2017). Three colony biofilms were combined and resuspended in 800 μL PBS supplemented with protease inhibitor cocktail and DNase I and passaged through a 23 g1 needle 10–15 times. Samples were sonicated with 20 impulses 4–6 times (Sonifier 250 (Branson), duty cycle 20%, output control 2) with cooling intermissions on ice between cycles. Disruption of cell clusters was verified under a microscope. After centrifugation for 20 min at 9000 *g* and 4°C supernatant was filtered (0.2 μm) and contained biofilm matrix while the pellet contained cells. Pellet was resuspended in same volume as before and sonicated by 2 × 20 impulses (duty cycle 20%, output control 2). Concentrations were determined with NanoDrop. For Western Blotting 100 μg samples were mixed with 4× SDS Loading dye and 10× Laemmli buffer (250 mM Tris, 1.92 M glycine, 1% SDS, pH 8.7).

#### 2.13.2 Isolation of Late Exponential Cells

For samples of exponential cultures, overnight cultures in LB medium (with respective antibiotics) were diluted to an OD_600_ of 0.1 and grown for 3 h until late exponential phase. 10 mL culture were harvested and stored at −20°C. Samples were resuspended in 4× SDS Loading dye, concentrated to an OD_600_ of 20, and sonicated 2× 20 impulses (Sonifier 250 (Branson), duty cycle 20%, output control 2) with cooling intermissions on ice between cycles. 10× Laemmli buffer were added to the samples.

#### 2.13.3 Immunoblotting

All samples were incubated for 10 min at 90°C, before loading into a 14% SDS PAGE containing 0.6% TCE. SDS PAGEs were run at 120 V and protein content was visualized in a ChemiDoc MD (Bio‐Rad). Proteins were transferred to a PVDF membrane (Bio‐Rad) activated with absolute ethanol. For BslA‐His detection the membrane was incubated with Penta‐His antibody (1:1000, ID: 34660, Qiagen) after blocking and further incubated with goat anti‐mouse secondary antibody HRP conjugate (1:5000, ID: 62‐6520, Invitrogen) before developing with Pierce ECL Western Blotting Substrate (Cat. No. 32109, Thermo Scientific). For AbrB detection the membrane was incubated with an AbrB antibody (1:5000, produced in rabbit, provided by Ulf Gerth) after blocking and further incubated with goat anti‐rabbit secondary antibody HRP conjugate (1:5000, ID: 65‐6120, Invitrogen) before developing with Pierce ECL Western Blotting Substrate (Cat. No. 32109, Thermo Scientific). Bioluminescence was detected with signal accumulation mode in ChemiDoc MD (Bio‐Rad).

For BslA‐His quantification of band intensities was performed with ImageJ gel analyser and normalized to band intensity of the WT168. Significances were calculated using one‐sample *t*‐test with *p*‐value ≤ 0.05 and results were depicted within the graphs (ns—non‐significant, and *p ≤ 0.05).

### 2.14 Statistical Analysis

Origin 2024b (Academic) was used to generate all graphs and conduct data analysis and statistical tests determining significance. Detailed description of statistical testing can be found within the methods.

## 3 Results

### 3.1 SamI Impacts Biofilm Development

The flotillin‐like proteins FloA and FloT are known to influence several developmental processes in *B. subtilis* . Loss of FloT impairs sporulation (Donovan and Bramkamp 2009; Yepes et al. 2012), while additional deletion of *floA* further reduces motility (Dempwolff et al. 2012), decreases protein secretion (Bach and Bramkamp 2013), and diminishes extracellular matrix production during biofilm formation (Yepes et al. 2012; Bach and Bramkamp 2013). In contrast, the role of the third SPFH protein, SamI, in *B. subtilis* physiology has remained unclear so far. Previous work from our group has shown that SamI, together with the membrane components SamG/H, is required for membrane localization of PspA (Scholz et al. 2021). However, the molecular mechanisms underlying the function of the SamGHI complex remain unclear. We found that deletion of *samI* in the laboratory strain *B. subtilis* WT168 results in a pronounced colony morphology phenotype. This observation provided a useful phenotypic readout for investigating SamI function and motivated us to use the WT168 background in this study. Although *B. subtilis* WT168 is not a classical model for biofilm formation, our aim was not to study biofilm development per se, but rather to elucidate the cellular function of SamI. We therefore chose the WT168 background, which also enabled us to build on previous work from our group investigating SPFH‐domain proteins in *B. subtilis* (Donovan and Bramkamp 2009; Bach and Bramkamp 2013, 2015; Zielińska et al. 2020; Scholz et al. 2021).

We first analysed the morphology of Δ*samI* biofilms grown on solid medium. Compared to the wild type strain (WT168), Δ*samI* colonies exhibited reduced structural complexity and smoother edges (Figure 1A). When biofilm formation at the liquid–air interface was tested, Δ*samI* was unable to form a surface‐covering pellicle (Figure 1A). A hallmark of the *B. subtilis* biofilm is its highly hydrophobic surface (Arnaouteli et al. 2016). Therefore, we quantified surface hydrophobicity by placing a water droplet on the biofilm and measured the contact angle. Wild type biofilms exhibited hydrophobic colony surfaces with a contact angle of 125.7° ± 6.58° (Figure 1B, Table S5). Δ*samI* biofilms lacked the characteristic hydrophobicity of wild type colony biofilms. Consequently, the contact angle dropped to zero (Figure 1B, Table S5). This phenotype was independent of the medium used, as similar effects were observed on different biofilm‐inducing media (Figure S1A,B, Table S5). Importantly, growth of Δ*samI* in shaking liquid cultures was indistinguishable from the wild type in both minimal and rich media (Figure S2A,B), indicating that SamI's role becomes relevant only upon initiation of biofilm differentiation. We further tested different background strains and observed that in the NCIB 3610 background the Δ*samI* mutant behaves similar in biofilm and pellicle development as the wild type (Figure S3A,B, Table S5), making the WT168 background the ideal strain to further investigate the function of SamI.

**FIGURE 1:**
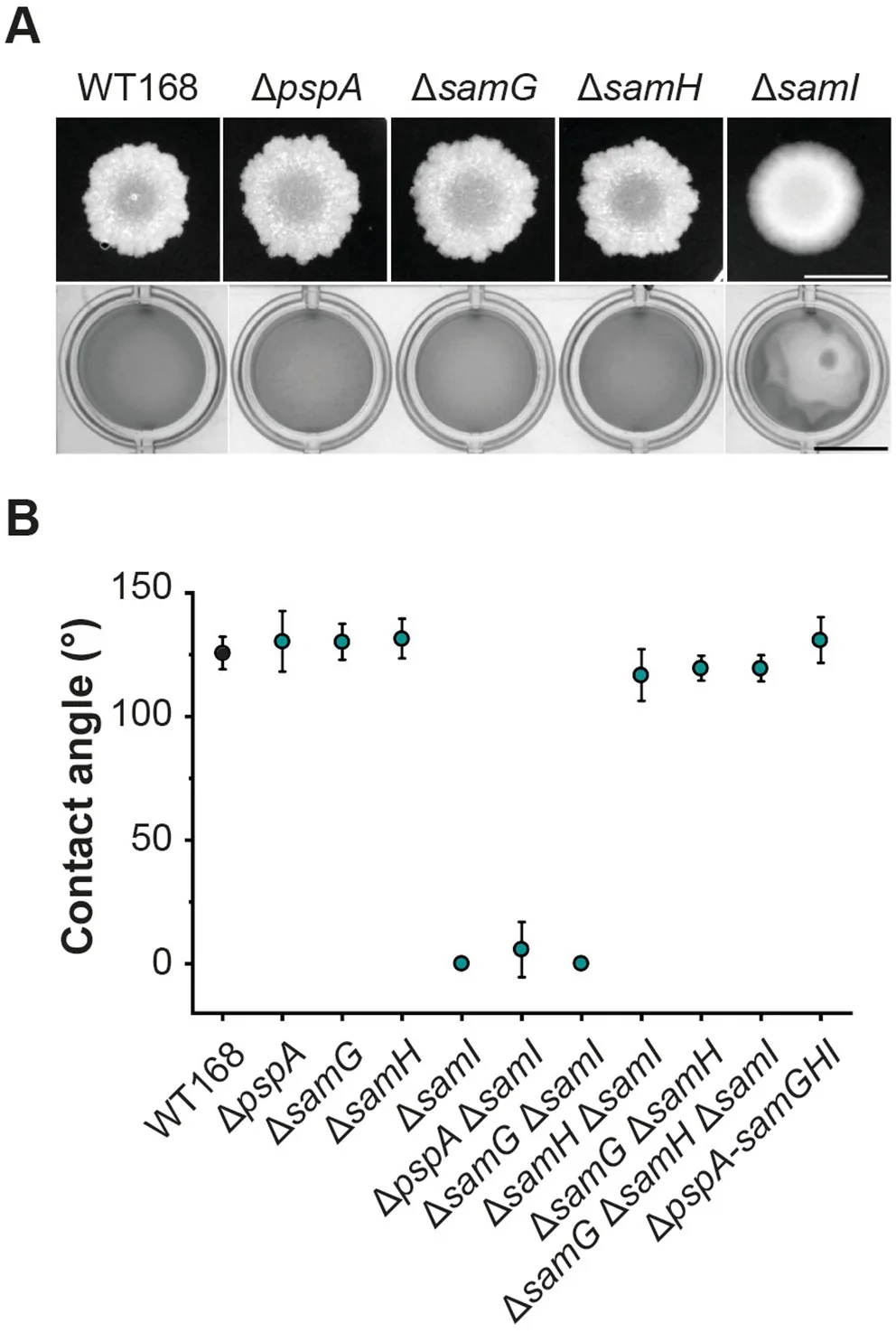
Deficient colony biofilm development of a *samI* mutant on liquid and solid medium. (A) Biofilm (upper panel, after 2 days) and pellicle growth (lower panel, after 7 days) of WT168 and single mutants of the *pspA‐samGHI* operon on LBGM medium (scale: 1 cm). (B) Hydrophobicity measurement of corresponding biofilms and additional double and triple deletions, as well as a full operon knock‐out (average and standard deviation was determined for three to nine independent replicates).

SamI is encoded in the *pspA*–*samGHI* operon together with PspA, SamG, and SamH. Because proteins of this operon have been reported to interact closely, we additionally tested single knock‐out strains and combinations of deletions of genes encoded in the *pspA*–*samGHI* operon. All further single mutants (Δ*pspA*, Δ*samG*, and Δ*samH*) revealed wild type like surface hydrophobicity, colony morphology, and pellicle development (Figure 1A,B, Table S5). Further, a double deletion of *samG* and *samH* also exhibited a hydrophobic surface. The hydrophilic surface of Δ*samI* was retained upon additional deletion of *pspA* or *samG* besides *samI*, whereas additional deletion of *samH* in the Δ*samI* background restored hydrophobicity, indicating that the Δ*samI* phenotype depends on the presence of SamH. Consistently, a triple deletion of *samG*, *samH*, and *samI*, as well as a complete operon knock‐out, both produced hydrophobic biofilms. These results suggest that the membrane protein SamH plays a major role during biofilm development, regulated by SamI.

To further explore the loss of biofilm hydrophobicity, we complemented the Δ*samI* strain by expressing *samI* or *samH* ectopically under an inducible promoter. Overexpression of either gene alone failed to restore hydrophobicity (Figure S4A, Table S5). Only when *samG* was additionally deleted, thus, removing a membrane anchor for SamI, induction of *samI* lead to a mild increase in hydrophobicity (Figure S4A). Since deletion of *samG* did not alter *samI* transcript levels (Figure S4B), this suggests that SamI functions as a regulator of the SamGH complex, thus leading to uncontrolled activity of the SamGH complex in absence of SamI during biofilm development. Together with the genetic interaction data, these findings indicate that absence of SamI could lead to unregulated activity of the membrane components SamGH.

We described before that deletion of *samI* gives rise to decreased membrane fluidity (Scholz et al. 2021). To test the influence of membrane fluidity on colony development, we also tested the effect of low concentrations of benzyl alcohol, a membrane‐fluidizing agent known to counteract membrane rigidification caused by loss of SPFH proteins (Bach and Bramkamp 2013; Zielińska et al. 2020; Álvarez‐Mena et al. 2025). In the wild type and in *floA* and *floT* single and double mutants, 0.05 to 0.1% benzyl alcohol supported formation of the hydrophobic coat, while higher concentrations of 0.2% benzyl alcohol led to higher variation in hydrophobicity, indicative of the growth inhibiting effect that benzyl alcohol can have of bacterial growth (Figure S5, Table S5) (Konopásek et al. 2000). In the Δ*samI* strain no impact of benzyl alcohol was observed, indicating that SamI's role in biofilm formation is not related to the membrane‐fluidity regulation typically associated with other SPFH proteins in *B. subtilis* .

To gain deeper insight into the morphological differences between wild type and Δ*samI* colony morphologies, we performed microscopic analyses. Brightfield images of resin sections of wild type biofilm, stained with Richardson solution, revealed a highly structured surface architecture (Figure 2A and Figure S6). An unstained layer of variable thickness was visible at the bottom of the biofilm after staining with Richardson solution (Figure 2A) corresponding to a layer of empty cell envelopes (Figure S6). In contrast, Δ*samI* biofilms exhibited a uniform, monotonous surface structure and an even layer of the empty cells at the bottom of the cross‐section. Higher‐resolution transmission electron micrographs of the biofilm cross‐sections revealed a thin, electron‐dense coat covering the wild type biofilm, which was absent in the Δ*samI* strain (Figure 2B). Scanning electron microscopy confirmed the presence and absence of this surface layer in wild type and *samI* mutant biofilms, respectively (Figure 2C). *B. subtilis* biofilms are known to be coated by a proteinaceous layer formed by BslA, which confers hydrophobic properties to the biofilm (Kobayashi and Iwano 2012; Hobley et al. 2013). Analysis of a Δ*bslA* biofilm revealed that Δ*bslA*, like Δ*samI*, exhibits a loss of architectural complexity (Figure 2A) and lacks the surface coat (Figure 2B,C), suggesting that the lack of this layer in Δ*samI* likely explains the observed loss of hydrophobicity.

**FIGURE 2:**
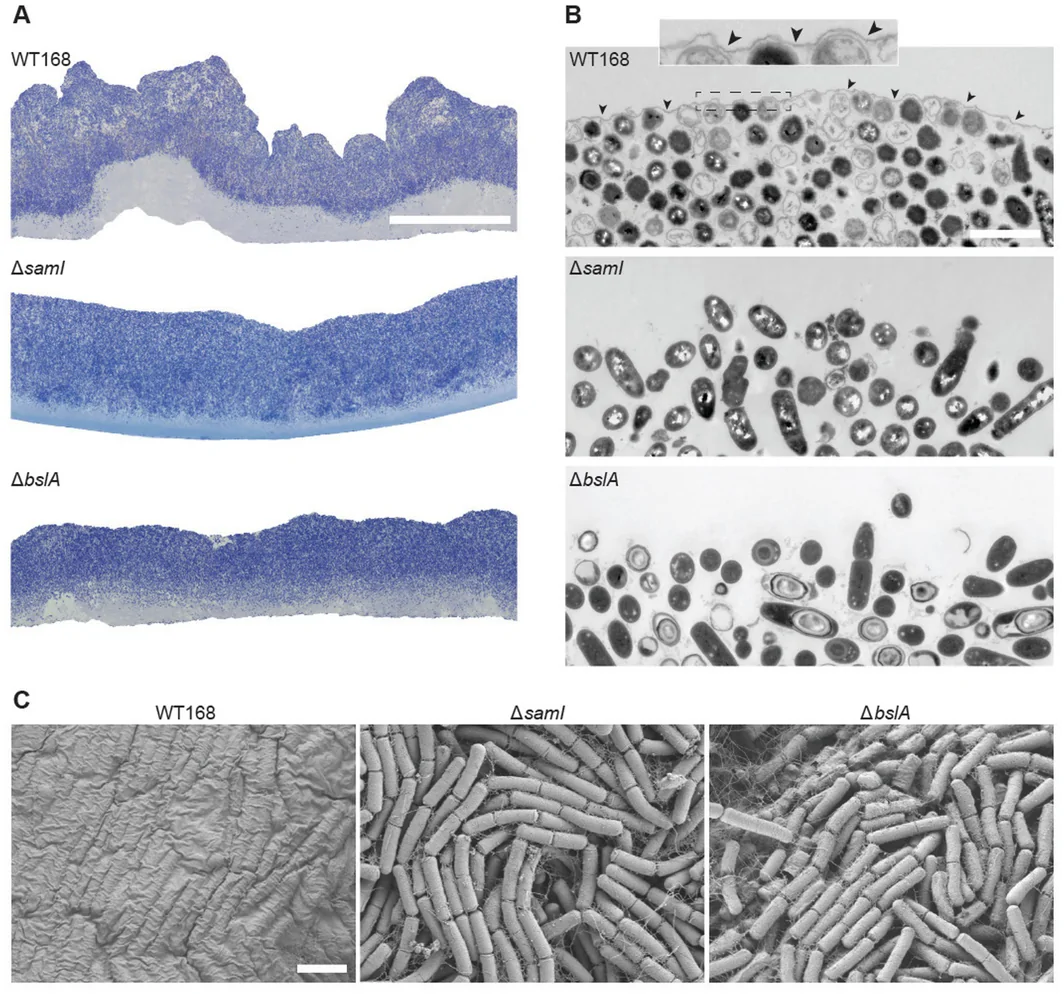
Δ*samI* leads to structural changes in colony biofilms. (A) Cross‐section morphology of WT168, Δ*samI*, and Δ*bslA* biofilms (scale: 100 μm). Brightfield images of 1 μm resin sections stained with Richardson solution. (B) TEM images of the biofilm surface in perpendicular 80 nm resin sections. The surface layer is present in WT168 (arrowheads) but missing in Δ*samI* and Δ*bslA* (scale: 2 μm). (C) SEM top‐view images of the biofilm surface in WT168, Δ*samI*, and Δ*bslA*. A continuous surface layer covers bacteria in WT168 (scale: 2 μm).

Our results indicate that SamI is important for maintaining cellular functions that support normal colony development in *B. subtilis* WT168. Loss of SamI causes a pronounced biofilm phenotype in this strain, including reduced architectural complexity and impaired formation of the hydrophobic BslA layer. Notably, this phenotype is absent in the wild type strain NCIB 3610, indicating that SamI is not intrinsically required for biofilm formation. Rather, loss of SamI appears to perturb cellular physiology in the WT168 background, compromising normal biofilm development. The membrane‐anchored SamGH proteins appear to contribute to this phenotype and may be functionally influenced by SamI.

### 3.2 Absence of SamI Leads to a Starvation Phenotype

To understand the effect SamI has during *B. subtilis* development we performed transcriptome analysis, comparing transcript levels between the wild type and Δ*samI* colonies. In total, 4242 protein coding sequences were detected. Of these transcripts, 49 and 112 (1.2% and 2.6%) had significantly increased or decreased levels, respectively (Figure 3A). Interestingly, the expression of the transition state regulator *abrB* was reduced in *samI* mutant biofilms. During the transition to biofilm development, increasing Spo0A~P levels repress *abrB* expression, thereby relieving the AbrB‐mediated repression of biofilm associated genes. A lower expression of the ICEBs1 genes was observed as well. ICEBs1 is an integrative and conjugative genetic element which is activated upon high cell densities and low nutrient availability or global DNA damage, leading to an induction of the SOS response (Auchtung et al. 2005). Further a reduction of genes involved in sporulation, motility, and chemotaxis, including a strong reduction of the flagellin *hag*, was observed. Additionally, significant changes in expression of gene clusters associated with metabolism and starvation were observed. This included changes in carbon metabolism, an increase in gene clusters involved in nitrogen and phosphate starvation, including genes responsible for phosphate scavenging and cell wall teichoic acid turnover, and a downregulation of genes associated with the methionine salvage pathway. Using reverse transcriptase qPCR, we investigated matrix genes and biofilm regulators further (Figure 3B). We confirmed a reduced transcription of *abrB* and found an increase of genes repressed by AbrB, including the matrix genes *bslA*, *epsA*, *tasA*, and the *lip* gene, encoding for an extracellular lipase. Transcription of other key biofilm regulators, including *spo0A*, *sinR*, and *degQ*, remained unchanged. Thus, the increased expression of matrix‐associated genes was not accompanied by transcriptional changes in these major regulatory genes. Overall, the transcriptome analysis indicates that loss of SamI triggers a nutrient‐starvation‐like response, accompanied by broad metabolic changes and increased transcription of matrix‐associated genes.

**FIGURE 3:**
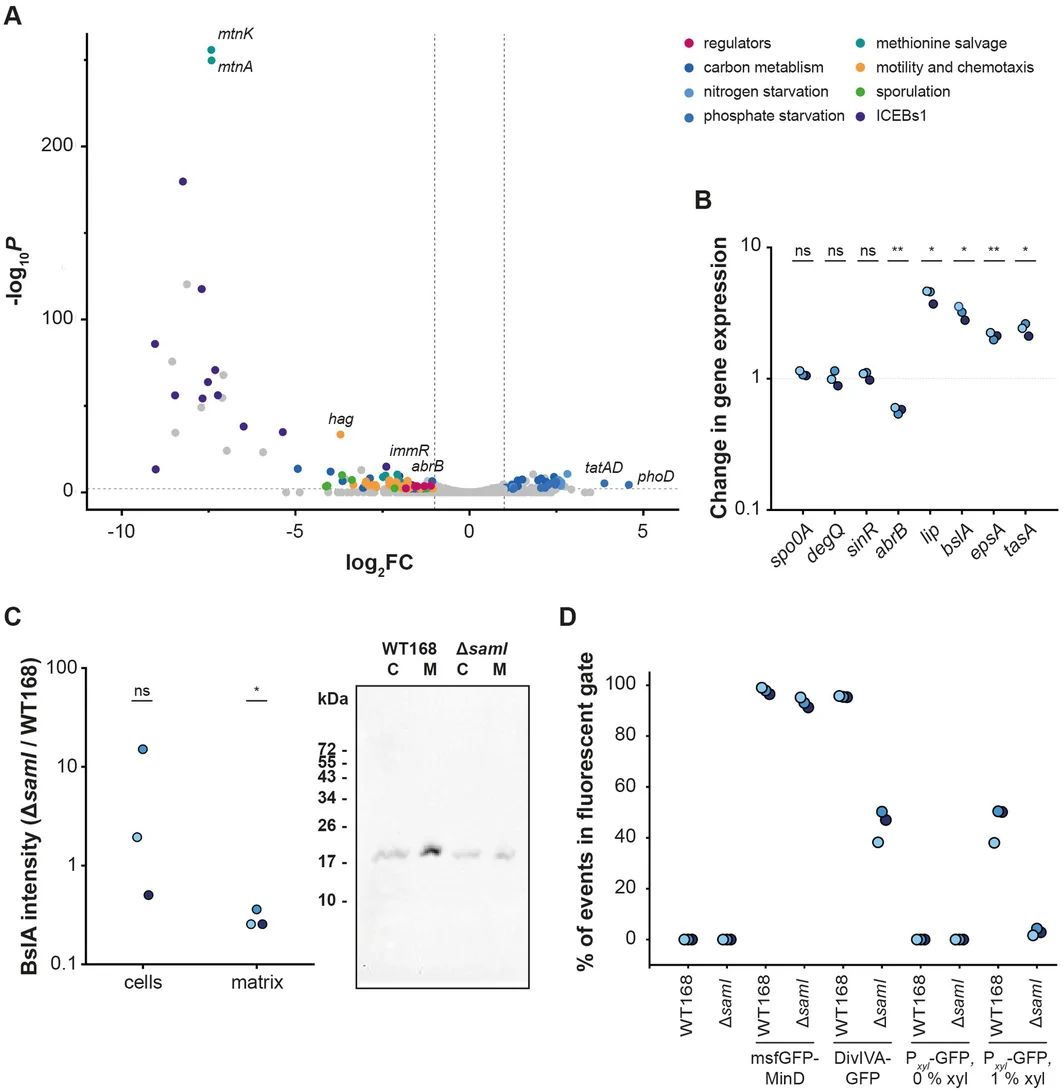
Transcriptional differences upon deletion of a *samI* mutant reveal changes in metabolism associated gene clusters and a reduction of BslA in the colony biofilm matrix. (A) Changes in gene expression upon *samI* deletion in a biofilm. RNAseq was performed with biofilms of WT168 and Δ*samI*. The −log_10_ adjusted *p*‐value (−log_10_P) was plotted against the log_2_ fold change (log_2_FC) in gene expression. Cut‐offs are depicted with dashed lines at log_2_FC ≥ 1, log_2_FC ≤ −1, and −Log_10_P ≤ 2. Significantly changed gene clusters are highlighted in different colours (see legend in upper right corner). (B) Changes in gene expression between WT168 and Δ*samI* in a biofilm measured with RT‐qPCR. Expression levels of regulators of biofilm development and matrix genes were determined (and normalized to levels of *divIVA*). Three independent replicates are depicted in different colours. Dashed line indicated no change in gene expression. Significance was calculated using one‐sample *t*‐test, *p*‐value ≤ 0.05. (C) Change in BslA levels between WT168 and Δ*samI* in a biofilm. BslA‐His intensity (20.2 kDa) was determined within a biofilm separated into the cells (C) and the matrix (M) and normalized to the WT168 (see left graph). Exemplary Western blot is shown on the right. Three independent replicates are depicted in different colours. Significance was calculated using one‐sample *t*‐test, *p*‐value ≤ 0.05. (D) Changes in fluorescence of msfGFP–MinD, DivIVA–GFP, and GFP expressed from P_xyl_ promoter in the absence (0% xylose) or presence (1% xylose) of xylose in WT168 and Δ*samI* colonies. The abundance of fluorescent events was determined by flow cytometry. Three independent replicates are depicted in different colours.

To investigate why colony biofilms are not formed properly in a *samI* mutant, despite the increase in matrix gene transcription, we investigated protein levels of BslA and AbrB. AbrB levels were determined in WT168 and Δ*samI* and revealed only very faint signal when extracted from biofilms (Figure S7B,C, original and unprocessed Western Blots and corresponding gels can be found in Figures S19–S22), while AbrB levels were detectable in late exponential phase (S7A). BslA levels were determined with a *bslA* poly‐histidine fusion integrated into the native locus of *bslA*. We tested if biofilm development was influenced by the additional His‐tag and confirmed that it had no effect on BslA's ability to form a hydrophobic coat during biofilm development (Figure S8A). Western blotting revealed that total BslA‐His levels were on average decreased in Δ*samI* compared to WT168 (Figure S8B, original and unprocessed Western Blots and corresponding gels can be found in Figures S13–S18). Further separation into a fraction containing the biofilm matrix and the cellular fraction revealed that BslA‐His was always reduced in the matrix of Δ*samI*, while BslA‐His levels within the cellular fraction showed strong variations (Figure 3C). Thus, higher *bslA* transcription did not consistently translate into efficient protein production and secretion giving rise to the observed biofilm phenotype of Δ*samI*.

The discrepancy between BslA transcript and protein levels, together with the metabolic changes observed in the RNA‐seq analysis, led us to investigate protein synthesis more broadly. We therefore analysed several reporter strains by flow cytometry, using fluorescent proteins as high‐throughput reporters of protein production. We selected genes for which RNA‐seq analysis revealed no significant differences in transcript levels between WT168 and Δ*samI*. These included two chromosomal fusion constructs, *msfgfp*‐*minD* and *divIVA*‐*gfp*, in which the fluorescent protein is encoded at the native locus, as well as a strain carrying *gfp* under the control of the inducible P_*xyl*_ promoter.

Flow‐cytometric analysis revealed a minor reduction in the proportion of fluorescent events for msfGFP‐MinD in the Δ*samI* background compared with WT168 (97.8% versus 93.2%, Figure 3D, Figure S9 for the original gating strategy). In contrast, the proportion of fluorescent events for DivIVA‐GFP was markedly reduced in Δ*samI* compared with WT168 (45.2% versus 95.5%, Figure 3D). Similarly, induction of P_*xyl*_‐*gfp* resulted in a strong increase in fluorescence in WT168 (from 0.02% to 46.2%), whereas only weak induction was observed in Δ*samI* (from 0.01% to 2.9%, Figure 3D). Together, these results indicate that protein production is broadly reduced during biofilm formation in the Δ*samI* background, despite comparable transcript levels, suggesting that loss of SamI affects gene expression at a post‐transcriptional or translational level.

In total, Δ*samI* leads to a decrease of transcription of *abrB* in colony biofilms, and thus an increase in matrix gene transcription. At the same time, a metabolic rewiring is observed in Δ*samI* resulting in a decreased protein translation and a decreased amount of BslA found in the matrix. Together, the transcriptome patterns and translational assays indicate that loss of SamI induces a global shift to a nutrient limitation response, where the actual accumulation of BslA protein in the matrix is reduced and in turn does not support correct biofilm maturation.

### 3.3 Absence of SamI Impacts SinR Pathway

Since transcriptional levels of matrix genes and protein levels of BslA were altered, we decided to further investigate the role of the biofilm regulators SinR and AbrB. For this we generated deletion mutants of *abrB* and *sinR* in the WT168 and Δ*samI* background. Additional Δ*abrB* had only a minor impact on biofilm morphology, leading to slightly smaller biofilms in both the WT168 and Δ*samI* background (see Figure S10A). Biofilm hydrophobicity remained unaltered in Δ*abrB* and Δ*abrB* Δ*samI* (see Figure S10B). Additional deletion of *sinR* on the other hand led to a strong change in biofilm morphology, leading to increased complexity and an increased size (see Figure S10A). But difference between the WT168 and Δ*samI* background remained, where Δ*sinR* Δ*samI* exhibited less structural complexity and an increased spreading compared to Δ*sinR*. Additionally, biofilm hydrophobicity was restored for the Δ*samI* background (see Figure S10B). Overall, the observed reversal of the biofilm phenotype of Δ*samI* in additional absence of *sinR* indicates that is due to misregulation of SinR activity leading to incomplete biofilm development.

Overall, the restoration of biofilm hydrophobicity and the partial reversal of the Δ*samI* biofilm phenotype upon deletion of *sinR* suggest that loss of SamI affects the SinR pathway, resulting in altered regulation of biofilm development. Suppression of the Δ*samI* phenotype by deletion of *sinR* further lends support to the notion that the observed phenotype is linked to loss of *samI* rather than to an unrelated second‐site mutation.

### 3.4 SamI Impacts Motility

During the transcriptional analysis, a reduction of motility‐associated genes was observed as well (Figure 3A). Therefore, we wanted to validate a potential implication on bacterial motility experimentally. Using a fluorescent fusion of FliM‐GFP, the number of flagella basal bodies in WT168 and Δ*samI* biofilms was determined (Figure 4A,B). In both the centre and the periphery of biofilms, a reduced number of FliM‐GFP foci was detected in Δ*samI*. While nearly 80% of WT168 cells contained five FliM‐GFP foci or more, only 30% of Δ*samI* cells contained that many foci in cells from the periphery of the biofilm. The number of cells with no foci more than doubled, from 14% to 39%, in the Δ*samI* mutant. In the centre of the biofilm, more than 80% of Δ*samI* cells contained no foci, and only a minor number of cells contained one or more foci. In WT168, approximately 25% of cells contained no FliM‐GFP foci. The remaining cells all contained a smaller number of foci, with the highest fraction having one focus (24%), followed by two foci (21%), and three foci (13%).

**FIGURE 4:**
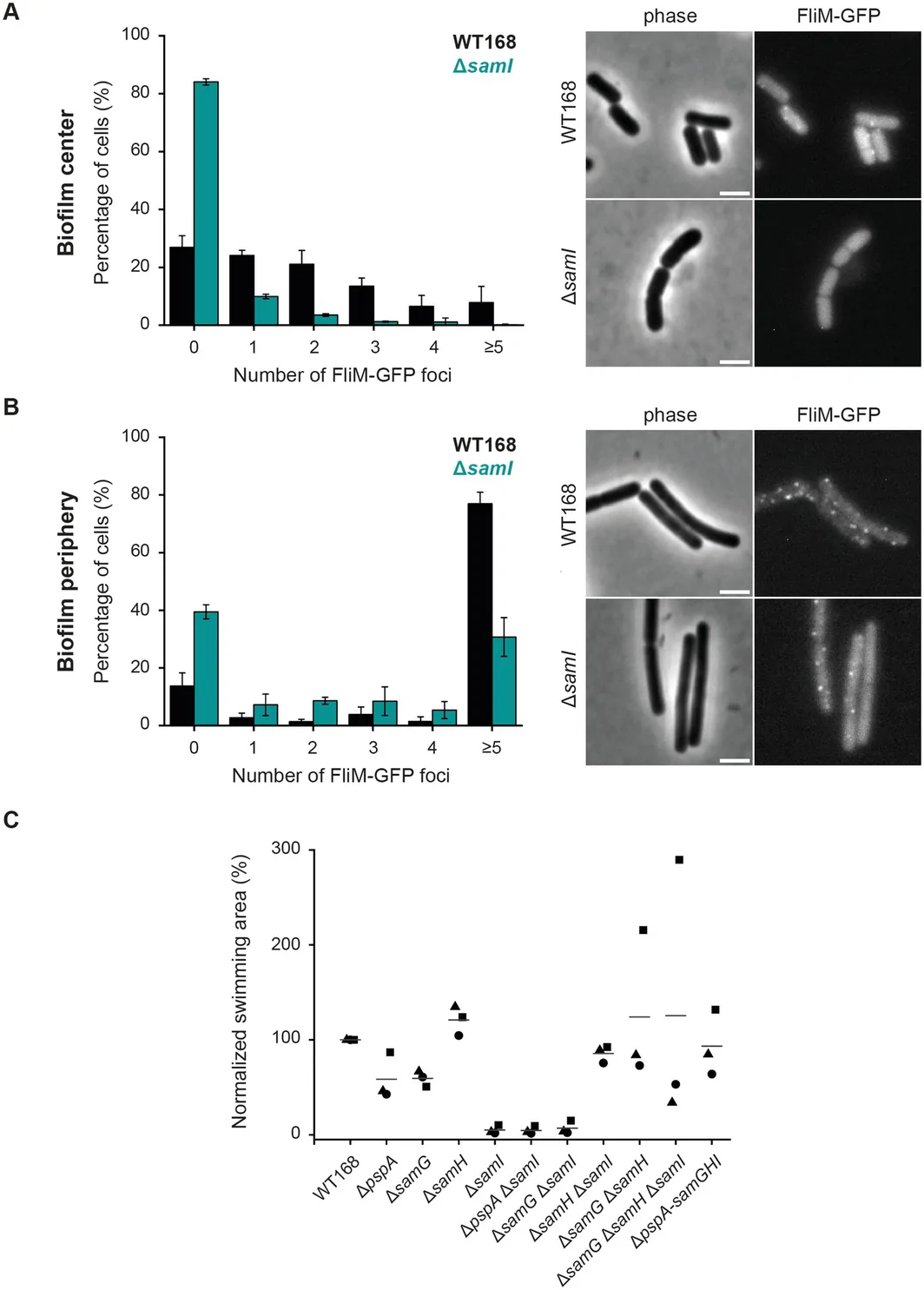
Loss of *samI* results in reduced motility. Number of FliM‐GFP foci in WT168 (black) and Δ*samI* (teal) were determined in central (A) and peripheral (B) areas of biofilms after 48 h (average and standard derivation of three independent replicates, *n* = 597–1285 cells per strain and area). Images of exemplary cell are shown on the right (scale: 2 μm). (C) Swimming progress after 10 h was determined for WT168 and all single mutants of the *pspA‐samGHI* operon and additional double‐, triple mutants and the full operon knock‐out. Swimming area was normalized to the swimming progress of WT168. Average normalized swimming area is depicted with a line and each independent replicate is visualized with a different shape (circle, rectangle, and triangle).

Therefore, we investigated SamI's influence on motility further. We here examined swimming motility, a unicellular mode of motility exhibited by highly flagellated cells (Kearns 2010). During swimming assays, Δ*samI* exhibited no swimming expansion until 10 h after inoculation compared to the WT168 (see Figure 4C). The strains Δ*pspA* Δ*samI* and Δ*samG* Δ*samI* phenocopied the behaviour of Δ*samI*, while the other deletion strains of the *pspA‐samGHI* operon showed swimming behaviour (see Figure 4C, Figures S11 and S12). Occasionally, Δ*samI* and Δ*samG* Δ*samI* exhibited swimming expansion after 20 h (Figures S11 and S12).

In summary, deletion of *samI* reduces swimming motility in *B. subtilis* . In line with this phenotype, a reduction in transcript levels of the motility and chemotaxis regulon was observed in Δ*samI* biofilms, and a reduced number of basal bodies was detected during biofilm development. Together, these results indicate that SamI is required for both the proper transcription of motility genes and the formation of flagellar structures.

## 4 Discussion

*B. subtilis* adapts to fluctuating environments by differentiating into distinct cell types capable of coping with specific stresses (Vlamakis et al. 2008; López and Kolter 2010; Qin et al. 2022). During exponential growth, a subset of cells adopts a motile phenotype, enabling dispersal into new niches (Kearns and Losick 2005). As cell density increases and nutrients become limiting, cells may differentiate into competent cells, cannibal cells, spores, or matrix‐producing cells that form protective biofilms (López et al. 2009; Mirouze and Dubnau 2013). Mature biofilms are heterogeneous, comprising motile, matrix‐producing, and sporulating subpopulations that collectively shape biofilm architecture (Vlamakis et al. 2008).

Here, we show that deletion of *samI*, which encodes an SPFH domain protein within the σ^W^‐regulated *pspA‐samGHI* operon, leads to systemic consequences during differentiation resulting in incomplete biofilm development: Deletion of *samI* produces smooth, non‐structured colonies with a hydrophilic surface (Figures 1 and 2). This phenotype is not observed upon single deletion of *pspA*, *samG*, *samH*, or the entire operon (Figure 1). Notably, co‐deletion of *samH* suppresses the Δ*samI* phenotype, and partial complementation of Δ*samI* requires deletion of *samG* (Figure 1 and S4). These observations indicate that the defect in hydrophobic biofilm formation does not result from simple loss of SamI function but is additionally influenced by the presence of SamH and modulated further by SamG. SamH contains a TPM domain, while SamI and SamG contain zinc finger motifs, respectively, that could facilitate regulatory functions (Scholz et al. 2021). Since the PspA‐SamGHI complex has previously been implicated as a membrane‐associated sensory complex (Scholz et al. 2021; Bramkamp and Scheffers 2023), SamG, SamH, and SamI may form a complex involved in signal perception and cellular adaption. Here, we observed that SamI restrains SamH activity and thereby allows downstream normal differentiation, resulting in development of a complex colony (Figure 5). In SamI's absence, SamH presumably becomes constitutively active, leading to metabolic rewiring in the population, resulting in incomplete biofilm development.

**FIGURE 5:**
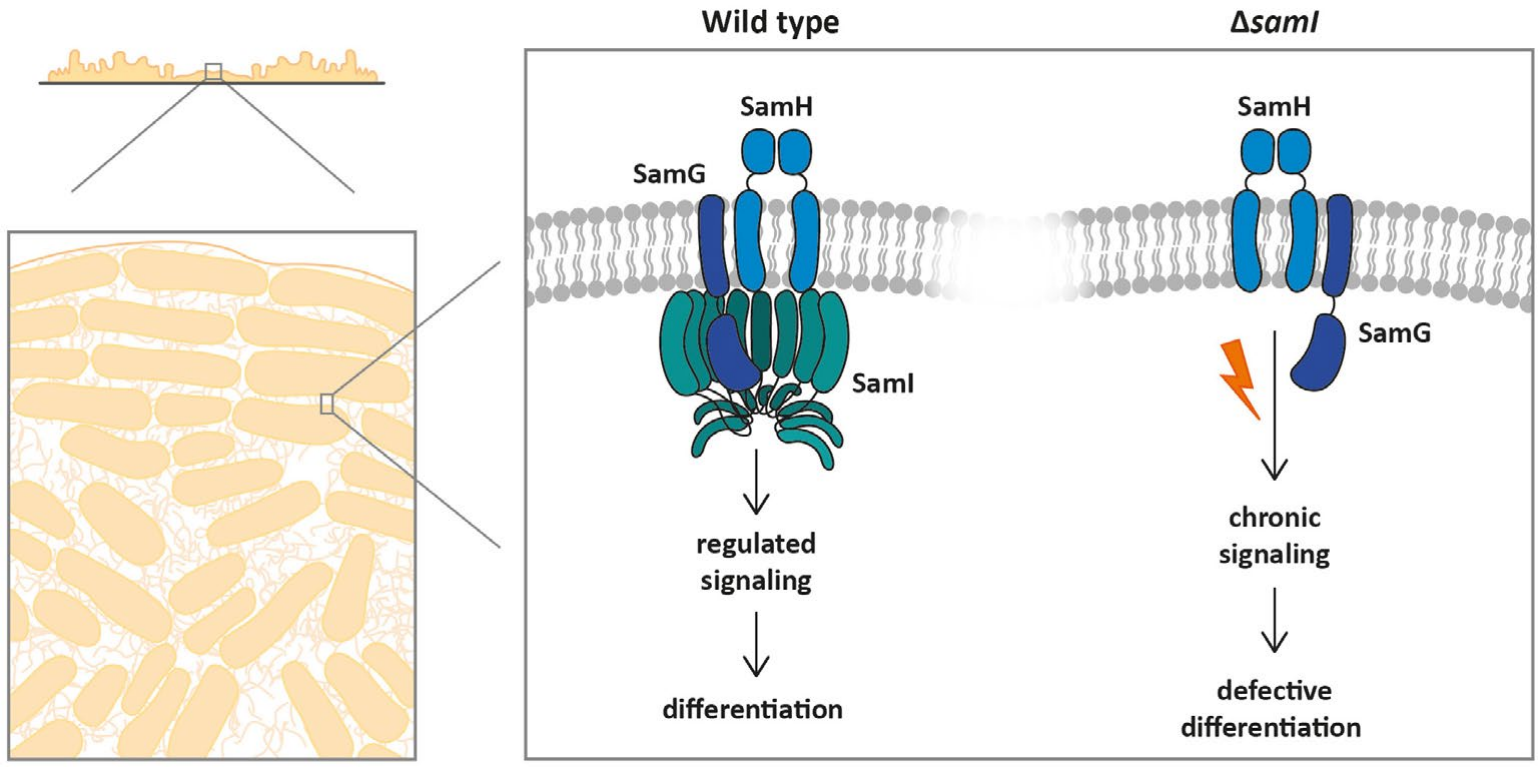
Schematic model of SamI's impact on colony biofilm development. In the wild type (left) SamI regulates the activity of SamH resulting in a normal stress response during biofilm development. In the absence of *samI* (right) SamH is constitutively active leading to a persistent signal resulting in metabolic changes that prevent BslA layer formation and flagellar basal body assembly. Oligomerization states are based on structure predictions from AlphaFold 3 predicting a monomer for SamG and a dimer for SamH. AlphaFold 3 predictions of SamI and unpublished data indicated a higher ordered oligomer.

Further, the biofilm‐deficient phenotype can be partially reversed by deletion of the biofilm repressor SinR (Figure S10), suggesting that loss of SamI alters regulation of the SinR‐controlled pathway. Together with the observed effects on cellular differentiation, these findings support a model in which SamI influences downstream regulatory processes through the SamGHI complex. However, further experiments are required to determine how the PspA‐SamGHI complex functions at the molecular level and whether its proposed role in membrane‐associated sensing and cellular adaptation is mechanistically linked to regulation of the SinR pathway.

This regulatory logic of a sensory component located at the cell membrane (SamH), which activity is modulated by a regulator (SamI), parallels the phage shock protein (Psp) system in *E. coli* (Kleerebezem et al. 1996; Jovanovic et al. 1996; Dworkin et al. 2000; Flores‐Kim and Darwin 2016; Popp et al. 2022). In that system, PspB and PspC form a membrane‐associated sensor, while PspA acts as a negative regulator that restrains the transcription factor PspF. Loss of PspA leads to constitutive stress signalling, mimicking the effect of chronic SamH activation in Δ*samI*. Although SamI and PspA differ structurally, a SPFH domain scaffold versus coiled‐coil regulator, the organizational principle is conserved: a membrane sensor complex is held in check by a regulatory subunit to prevent persistent stress responses. The *E. coli* PspA is, however, not only a regulator of the phage shock system, but also the executing module. Upon envelope stress perception through PspB and PspC (Kleerebezem et al. 1996; Flores‐Kim and Darwin 2016) PspA oligomerises into larger complexes, thought to protect membrane integrity (Engl et al. 2009). Thus, in the firmicute system the regulation of the sensory module and the effector complex have been separated into two proteins, PspA and SamI, respectively.

Further conceptual parallels exist between SamI/SamH and the LiaRS two‐component system in *B. subtilis* , which responds to antibiotic and cell envelope induced stress (Radeck et al. 2017). In the Lia system, LiaF inhibits the sensor kinase LiaS under non‐stress conditions, leaving LiaS phosphatase activity intact, preventing constitutive activation of the LiaRS regulon (Jordan et al. 2006). Only upon envelope perturbation is this inhibition relieved, triggering an adaptive stress response. This mirrors the function of SamI, which restrains SamH signalling under normal conditions. Loss of SamI produces unrestrained SamH activity, analogous to removal of LiaF, resulting in chronic signalling and maladaptive cellular responses. Together, these examples illustrate a conserved regulatory principle in *B. subtilis* : membrane‐associated sensor complexes require inhibitory “brake” proteins to prevent constant signalling, whether in the context of biofilm development (SamI/SamH) or antibiotic‐induced envelope stress (LiaF/LiaRS).

Members of the SPFH domain family have been shown to frequently function as scaffolds that spatially organize and regulate membrane‐associated complexes (Bramkamp and López 2015; Willdigg and Helmann 2021; Bramkamp and Scheffers 2023). Examples include HflK/C regulating the FtsH protease in *E. coli* , the SPFH‐NfeD complex QmcA‐YbbJ in *E. coli* , and the stomatin‐like protein StlP controlling cellulose synthase complexes in *Streptomyces coelicolor* (Ma et al. 2022; Qiao et al. 2022; Tan et al. 2024; Zhong et al. 2025). SamI likely acts in a similar manner, modulating SamH signalling and preventing chronic activation during multicellular development.

Importantly, the regulation of membrane spanning signalling proteins mediated by SPFH domain protein is also conserved in eukaryotes. Flotillins and stomatins have been implicated in organizing membrane microdomains in eukaryotic cells (Langhorst et al. 2005; Browman et al. 2007; Lapatsina et al. 2012; Yokoyama and Matsui 2020). Flotillin‐1 and ‐2 regulate the levels of CD14 on macrophages, which recognizes lipopolysaccharides (LPS) thus activating several Toll‐like receptor (TLR) signalling pathways, including TLR4 of the innate immune response (Matveichuk et al. 2024). Also, Flotillin‐1 positively regulates endosomal TLR3 signalling in endothelial cells by promoting ligand transport and caveolae‐dependent receptor activation, thereby enabling inflammatory responses (Fork et al. 2014). These examples reveal the universal role of SPFH domain proteins in signalling pathways in pro‐ and eukaryotes.

Loss of SamI in *B. subtilis* triggers profound physiological consequences. Δ*samI* biofilms alter transcription of metabolism associated gene clusters, leading to a reduced translational capacity (Figure 3). This in turn drastically reduced secretion of the matrix protein BslA resulting in loss of biofilm architecture (Figures 1 and 2, Figures S6, S8 and S9). Another consequence of the starvation phenotype is the downregulation of motility associated genes. Consequently, FliM‐GFP basal bodies were found to be reduced, and swimming ability was impaired (Figure 4). Further evidence for a starvation phenotype that affects cellular energy levels is the downregulation of ICEBs1 genes in Δ*samI* biofilms, indicating that horizontal gene transfer is suppressed under these conditions (Figure 3). This is likely a consequence of the starvation‐like metabolic state observed in Δ*samI* cells. Maintenance of ICEBs1 within a population is an energy‐intensive process requiring integration, excision, replication, and translocation of ICEBs1 (Auchtung et al. 2016). While replication can include multiple rounds of replication per donor, translocation is facilitated by a Type 4 secretion system (T4SS) including the putative ATPases ConQ and ConE, responsible for coupling of the ICEBs1 to the T4SS and DNA transfer, respectively (Lee et al. 2010; Berkmen et al. 2010; Auchtung et al. 2016; Murthy et al. 2023). Under nutrient‐limited or low‐energy conditions, cells likely prioritize essential survival functions over horizontal gene transfer, leading to reduced expression of ICEBs1 genes. These findings highlight how the metabolic consequences of chronic SamH activity in the absence of SamI can directly impact cellular programs beyond biofilm formation, including adaptive gene exchange. Since RNAseq experiments of the biofilms was only performed at one timepoint, differences in development before that timepoint remain unknown. Furthermore, some of the gene clusters here observed with changed expression (e.g., ICEBs1, sporulation, different metabolism clusters, Figure 3) were not investigated further. Considering the observed contradicting levels of *blsA* and BslA as well as transcript and protein levels of the reporter strains tested during flow cytometry (compare Figure 3B vs. Figure 3C and RNAseq data set with Figure 3D) this raises the question of how other changes in RNA transcript levels will correspond to protein levels. But these questions will have to be analysed in further studies.

## 5 Conclusion

In conclusion, SamI functions as a regulator of the SamGH stress‐sensing and signalling complex, restraining SamH‐mediated activity and thereby supporting appropriate downstream cellular responses. The biofilm phenotype observed upon loss of SamI in *B. subtilis* WT168 therefore likely represents a consequence of dysregulated SamGH signalling rather than a biofilm‐specific function of SamI. By preventing unrestrained SamH activity, SamI ensures correct cellular responses during growth on surfaces (Figure 5). This is important at the population level to allow the fine‐tuned differentiation of different developmental pathways. In turn, unregulated signal transduction leads to misregulation of the signal cascades governing these programs. The analogy with the *E. coli* Psp system highlights a conserved design principle in bacteria: sensor complexes require regulatory subunits to avoid maladaptive chronic stress signalling. In *B. subtilis* , SamI exemplifies this regulatory principle, enabling cells to integrate stress perception with multicellular development.

## Author Contributions

**Sarah S. M. Baur:** investigation, writing – original draft, methodology, validation, visualization, writing – review and editing, formal analysis. **Urška Repnik:** investigation, writing – review and editing, methodology, validation, formal analysis, resources. **Tobias Busche:** investigation, writing – review and editing, methodology, validation, formal analysis, resources. **Louisa Rau:** investigation, writing – review and editing. **Alisa Mondry:** investigation, writing – review and editing. **Marc Bramkamp:** conceptualization, investigation, funding acquisition, writing – original draft, methodology, writing – review and editing, project administration, supervision, resources.

## Funding

This work was supported by Deutsche Forschungsgemeinschaft, BR2915/7‐1, INST 257/688‐1 (FUGG), INST 257/475‐3, CRC 1182 project Z2.

## Conflicts of Interest

The authors declare no conflicts of interest.

## Supporting information


**Data S1:** Supporting information.
**Figure S1:** Deficient biofilm phenotype of Δ*samI* is independent of medium. (A) Biofilm development of WT168 and Δ*samI* on LBGM (after 48 h) and Msgg agar (after 72 h, scale: 1 cm). (B) Surface hydrophobicity of Δ*samI* biofilms is deficient on full (LBGM) and minimal medium (MSgg). Average contact angle and standard derivation was determined in three independent replicates.
**Figure S2:** emi70467‐sup‐0001‐Supinfo.pdf. *SamI* mutant grows indistinguishable from WT168 in liquid cultures. Growth curves of WT168 (black) and Δ*samI* (teal) in full (LB) medium (A) and minimal (MD) medium (B) over a time course of 24 h. Average and standard derivation are depicted of three independent replicates.
**Figure S3:** Deficient biofilm phenotype of Δ*samI* only observed in WT168 background. (A) Biofilm and pellicle development of wildtype and Δ*samI* in WT168 and NCIB 3610 background. LBGM agar (upper panel) was used to observe biofilm development, and pellicles were grown for 7 days in liquid LBGM (lower panel) (scale: 1 cm). (B) Deficient surface hydrophobicity of Δ*samI* is only observed in WT168 background. Average contact angle and standard derivation was determined in three independent replicates.
**Figure S4:** Absence of *samG* leads to partial complementation of *samI*. (A) Surface hydrophobicity in Δ*samI* can only be restored in complementation strains when SamI membrane anchor, SamG, is missing. Biofilms were grown on LBGM supplemented with 1 mM IPTG. Contact angle and standard derivation was determined of three to six independent replicates of WT168, Δ*samI*, and its complementation strains. (B) Transcript level of *samI* in the corresponding strains normalized to level of *samI* in WT168. Dashed line indicated no change in gene expression.
**Figure S5:** Membrane fluidization does not improve Δ*samI's* biofilm phenotype. Surface hydrophobicity of SPFH mutants of LBGM supplemented with 0% to 0.2% benzyl alcohol. Contact angle and standard derivation were determined of three independent replicates.
**Figure S6:** Biofilms of WT168 and Δ*samI* have a layer of empty cells at the bottom of the biofilm. TEM images of showing an overview over cross section of WT168 (left) and Δ*samI* (right) biofilms. On the bottom of the biofilms a layer of empty cell envelopes is visible (scale: 10 μm).
**Figure S7:** AbrB levels in WT168 and Δ*samI* exponential cultures and biofilms. AbrB levels (10.6 kDa) were determined for three independent biological replicates in late exponential cultures (A) and of cells isolated from biofilms (B and C). The lane for the ladder is marked with L. For the Western Blot of biofilm samples (B) the area around 10 kDa was adjusted in brightness and contrast to enhance the very faint signal detected during western blotting (C).
**Figure S8:** BslA‐His does not influence biofilm hydrophobicity and Δ*samI* exhibit reduced BslA‐His levels. (A) Surface hydrophobicity of WT168 and Δ*samI* is not impacted by His tagged BslA. Contact angle and standard derivation were determined of three independent replicates. (B) Change in total BslA‐His levels between WT168 and Δ*samI* isolated from biofilms. Three independent replicates are depicted in different colours.
**Figure S9:** Original blots of flow cytometric analysis of reporter strains exhibiting reduced abundance of fluorescent events in Δ*samI* background compared to WT168. WT168 and Δ*samI* were used as control to determine the placement of the gates (first row). P4 gate (black, corresponds to fluorescent population in WT168), P3 gate (grey, corresponds to non‐fluorescent population in WT168), P2 gate (dark turquoise, corresponds to fluorescent population in Δ*samI*) and P1 gate (bright turquoise, corresponds to non‐fluorescent population in Δ*samI*). msfGFP‐MinD (second row), DivIVA‐GFP (third row), P_*xyl*_‐GFP (0% xylose), (fourth row), and GFP (1% xylose, (fifth row)).
**Figure S10:** Biofilm phenotype of Δ*samI* can be partially recovered upon additional deletion of Δ*sinR*. (A) Biofilm development of WT168 and Δ*samI* was determined upon additional deletion of Δ*abrB and* Δ*sinR* on LBGM agar (scale: 1 cm). (B) Surface hydrophobicity can be completely recovered upon additional loss of *sinR*. Average contact angle and standard derivation was determined in three independent replicates.
**Figure S11:** Swimming progress of WT168, single, double, triple mutants, and the full operon knock‐out of the *pspA‐samGHI* operon. Swimming progress was observed over a time course of 20 h and average area (in cm^2^) and standard derivation were determined of three independent replicates.
**Figure S12:** Original images of swimming progress of one exemplary replicate. Swimming progress was determined for the WT168, single, double, triple mutants, and the full operon knock‐out of the *pspA‐samGHI* operon over a time course of 20 h. Swimming assays were performed in standard plates (diameter: 92 mm).
**Figure S13:** Original unprocessed Western Blot 1, used for and shown in Figure 3C. Cellular (C) and matrix (M) fraction isolated from biofilms of WT168 and Δ*samI* were analysed with Western Blotting for three independent replicates (first replicate shown here). The lanes for the ladder are marked with L. The corners of the Western Blot are marked with small red corners. Areas that were analysed are inside the blue box. The BslA‐His signal (20.2 kDa) is highlighted with an arrow. Western Blot 1 corresponds to TCE strained gel 1.
**Figure S14:** Original unprocessed TCE stained gel 1, used for Figure 3C. Cellular (C) and matrix (M) fraction isolated from biofilms of WT168 and Δ*samI* were analysed with Western Blotting for three independent replicates (TCE stained gel of first replicate shown here). The lane for the ladder are marked with L. For better visibility the annotation of the ladder is shown on the left side beside the gel. Areas of interest are inside the blue box. The corresponding localization of the corners of the Western Blot membrane have been visualized in the TCE stained gels with small red corners. TCE strained gel 1 corresponds to Western Blot 1.
**Figure S15:** Original unprocessed Western Blot 2, used for Figure 3C. Cellular (C) and matrix (M) fraction isolated from biofilms of WT168 and Δ*samI* were analysed with Western Blotting for three independent replicates (second replicate shown here). The lane for the ladder is marked with L. The corners of the Western Blot are marked with small red corners. Areas that were analysed are inside the blue box. The BslA‐His signal (20.2 kDa) is highlighted with an arrow. Western Blot 2 corresponds to TCE strained gel 3.
**Figure S16:** Original unprocessed TCE stained gel 2, used for Figure 3C. Cellular (C) and matrix (M) fraction isolated from biofilms of WT168 and Δ*samI* were analysed with Western Blotting for three independent replicates (TCE stained gel of second replicate shown here). The lane for the ladder is marked with L. For better visibility the annotation of the ladder is shown on the left side beside the gel. Areas of interest are inside the blue box. The corresponding localization of the corners of the Western Blot membrane have been visualized in the TCE stained gels with small red corners. TCE strained gel 2 corresponds to Western Blot 2.
**Figure S17:** Original unprocessed Western Blot 3, used for Figure 3C. Cellular (C) and matrix (M) fraction isolated from biofilms of WT168 and Δ*samI* were analysed with Western Blotting for three independent replicates (third replicate shown here). The lane for the ladder is marked with L. The corners of the Western Blot are marked with small red corners. Areas that were analysed are inside the blue box. The BslA‐His signal (20.2 kDa) is highlighted with an arrow. Western Blot 3 corresponds to TCE strained gel 3.
**Figure S18:** Original unprocessed TCE stained gel 3, used for Figure 3C. Cellular (C) and matrix (M) fraction isolated from biofilms of WT168 and Δ*samI* were analysed with Western Blotting for three independent replicates (TCE stained gel of third replicate shown here). The lane for the ladder is marked with L. For better visibility the annotation of the ladder is shown on the left side beside the gel. Areas of interest are inside the blue box. The corresponding localization of the corners of the Western Blot membrane have been visualized in the TCE stained gels with small red corners. TCE strained gel 3 corresponds to Western Blot 3.
**Figure S19:** Original unprocessed Western Blot 4, used for Figure S8A. Three independent replicates (1 to 3) of exponentially growing WT168 and Δ*samI* cultures were analysed with Western Blotting. The lane for the ladder is marked with L. The corners of the Western Blot are marked with small red corners. The AbrB signal (10.6 kDa) is highlighted with an arrow. Western Blot 4 corresponds to TCE strained gel 4.
**Figure S20:** Original unprocessed TCE stained gel 4, used for Figure S8A. Three independent replicates (1 to 3) of exponentially growing WT168 and Δ*samI* cultures were analysed with Western Blotting (TCE stained gel of western blot 4 shown here). The lane for the ladder is marked with L. For better visibility the annotation of the ladder is shown on the left side beside the gel. The corresponding localization of the corners of the Western Blot membrane have been visualized in the TCE stained gels with small red corners. TCE strained gel 4 corresponds to Western Blot 4.
**Figure S21:** Original unprocessed Western Blot 5, used for Figure S8B,C. Three independent replicates (1 to 3) of WT168 and Δ*samI* biofilms were analysed with Western Blotting. The lane for the ladder is marked with L. The corners of the Western Blot are marked with small red corners. The region where AbrB signal (10.6 kDa) was detected upon editing (see Figure S8 for more information) is highlighted with an arrow. Western Blot 5 corresponds to TCE strained gel 5.
**Figure S22:** Original unprocessed TCE stained gel 5, used for Figure S8B,C. Three independent replicates (1 to 3) of WT168 and Δ*samI* biofilms were analysed with Western Blotting (TCE stained gel of western blot 5 shown here). The lane for the ladder is marked with L. For better visibility the annotation of the ladder is shown on the left side beside the gel. The corresponding localization of the corners of the Western Blot membrane have been visualized in the TCE stained gels with small red corners. TCE strained gel 5 corresponds to Western Blot 5.
**Table S1:** emi70467‐sup‐0001‐Supinfo.pdf. *Bacillus subtilis* strains used in this study.
**Table S2:** emi70467‐sup‐0001‐Supinfo.pdf. *Escherichia coli* strains used in this study.
**Table S3:** Plasmids used in this study.
**Table S4:** Oligonucleotides used in this study.
**Table S5:** Average contact angles and corresponding standard derivation (SD).

## Data Availability

The data that support the findings of this study are openly available in Gene Expression Omnibus at https://www.ncbi.nlm.nih.gov/, reference number GSE327651.
