# Supplementary material for: An SPFH Protein Couples Membrane Stress to Differentiation in Bacillus subtilis
Source: Environ Microbiol. 2026 Oct 27;28(10):e70467. doi: 10.1111/1462-2920.70467 (PMC13713150; doi:10.1111/1462-2920.70467)
Supplement: Supplementary file 1 [file EMI-28-e70467-s001.pdf]

Supplementary Material

An SPFH Protein Couples Membrane Stress to Differentiation in *Bacillus subtilis*

Sarah S M Baur[1], Urška Repnik[2], Tobias Busche[3], Louisa Rau[1], Alisa Mondry[1,4], and Marc Bramkamp[1,2*]

[1] Christian-Albrechts-Universität zu Kiel, Mathematisch-Naturwissenschaftliche Fakultät, Institut für Allgemeine Mikrobiologie, Am Botanischen Garten 1-9, 24118 Kiel, Germany

[2] Christian-Albrechts-Universität zu Kiel, Mathematisch-Naturwissenschaftliche Fakultät, Zentrale Mikroskopie, Am Botanischen Garten 5, 24118 Kiel, Germany

[3] Bielefeld University, Medical School OWL & Center for Biotechnology (CeBiTec), Universitätsstraße 25, 33615 Bielefeld, Germany

[4] MRC Laboratory of Molecular Biology, Cambridge CB2 0QH, United Kingdom

Keywords: SPFH, flotillin, membrane, biofilm, BslA, starvation, motility

**Correspondence:* Marc Bramkamp, Institute for General Microbiology, Kiel University, Germany. E-mail: bramkamp@ifam.uni-kiel.de

1. Supplementary figures

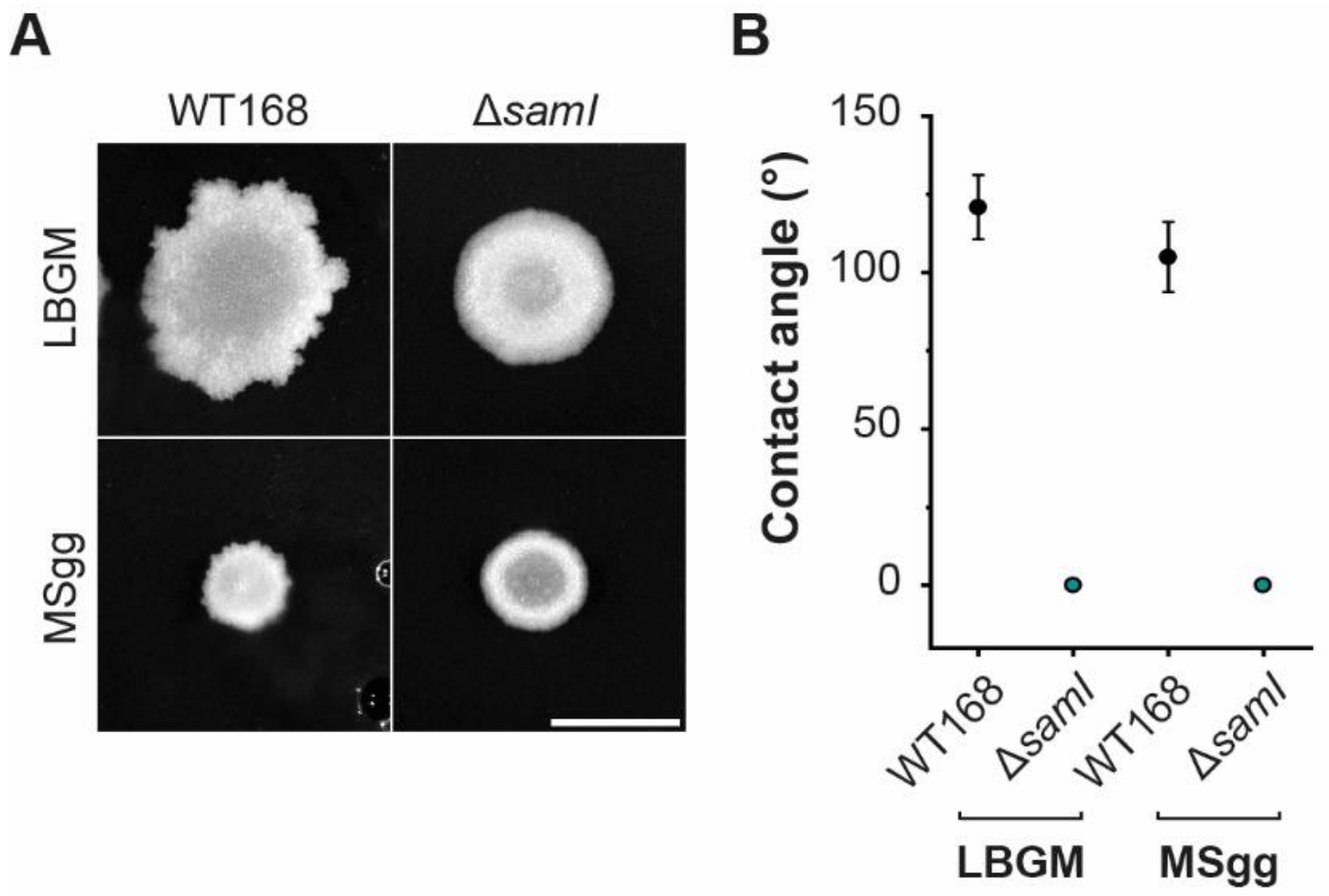

Supplementary Figure S1: Deficient biofilm phenotype of Δ*samI* is independent of medium. (A) Biofilm development of WT168 and Δ*samI* on LBGM (after 48 h) and Msgg agar (after 72 h, scale: 1 cm). (B) Surface hydrophobicity of Δ*samI* biofilms is deficient on full (LBGM) and minimal medium (MSgg). Average contact angle and standard derivation was determined in three independent replicates.

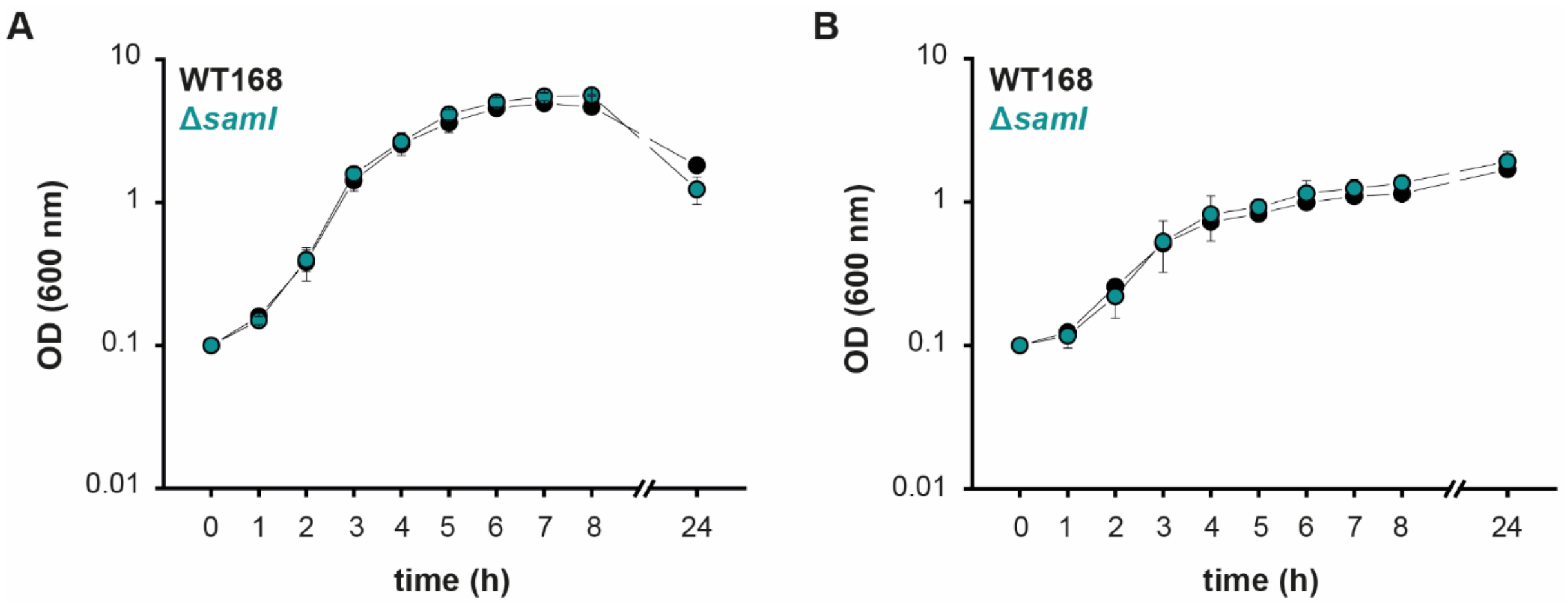

Supplementary Figure S2: *SamI* mutant grows indistinguishable from WT168 in liquid cultures. Growth curves of WT168 (black) and Δ*samI* (teal) in full (LB) medium (A) and minimal (MD) medium (B) over a time course of 24 h. Average and standard derivation are depicted of three independent replicates.

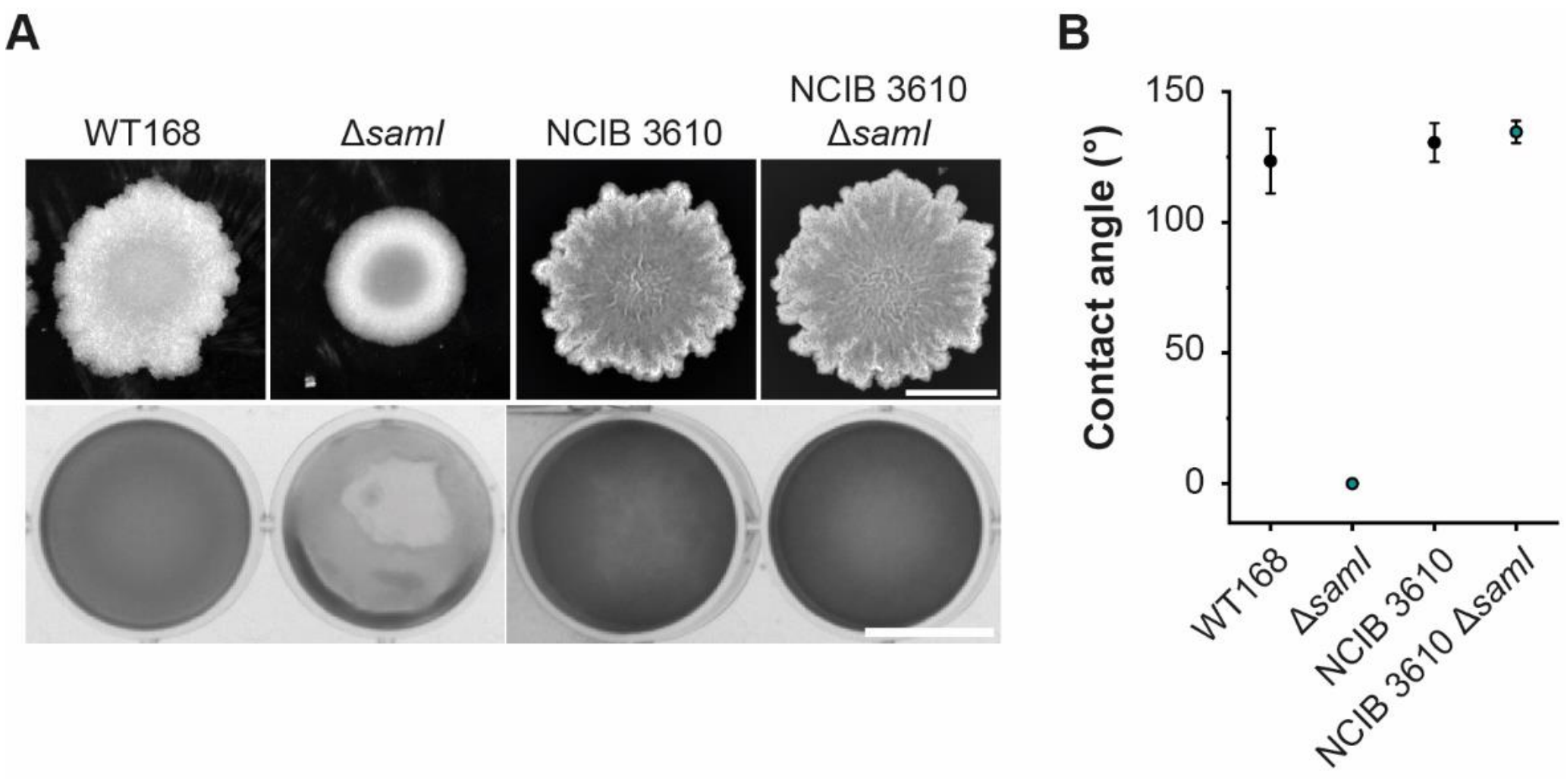

Supplementary Figure S3: Deficient biofilm phenotype of Δ*samI* only observed in WT168 background. (A) Biofilm and pellicle development of wildtype and Δ*samI* in WT168 and NCIB 3610 background. LBGM agar (upper panel) was used to observe biofilm development, and pellicles were grown for 7 days in liquid LBGM (lower panel) (scale: 1 cm). (B) Deficient surface hydrophobicity of Δ*samI* is only observed in WT168 background. Average contact angle and standard derivation was determined in three independent replicates.

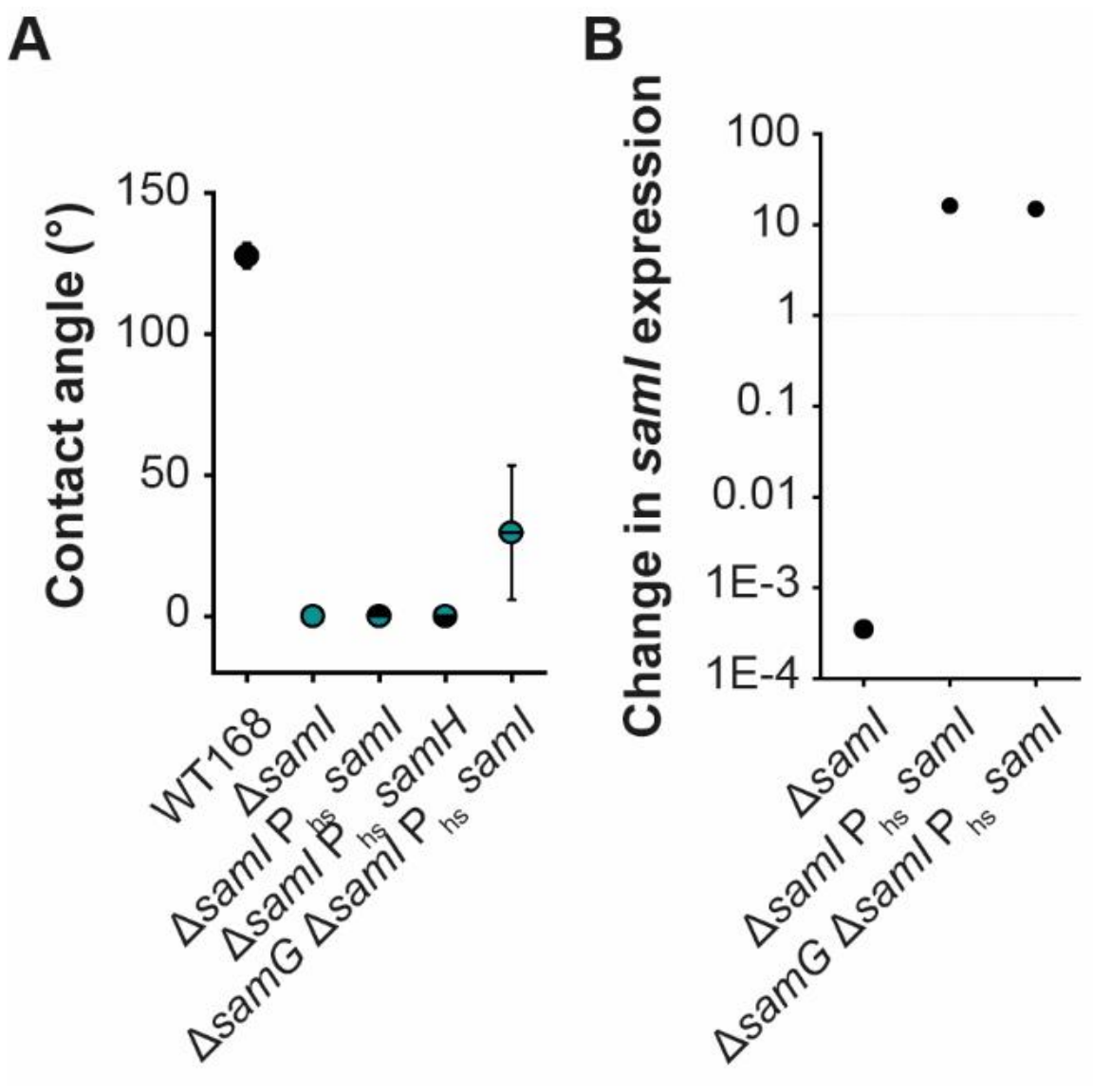

Supplementary Figure S4: Absence of *samG* leads to partial complementation of *samI*. (A) Surface hydrophobicity in Δ*samI* can only be restored in complementation strains when SamI membrane anchor, SamG, is missing. Biofilms were grown on LBGM supplemented with 1 mM IPTG. Contact angle and standard derivation was determined of three to six independent replicates of WT168, Δ*samI*, and its complementation strains. (B) Transcript level of *samI* in the corresponding strains normalized to level of *samI* in WT168. Dashed line indicated no change in gene expression.

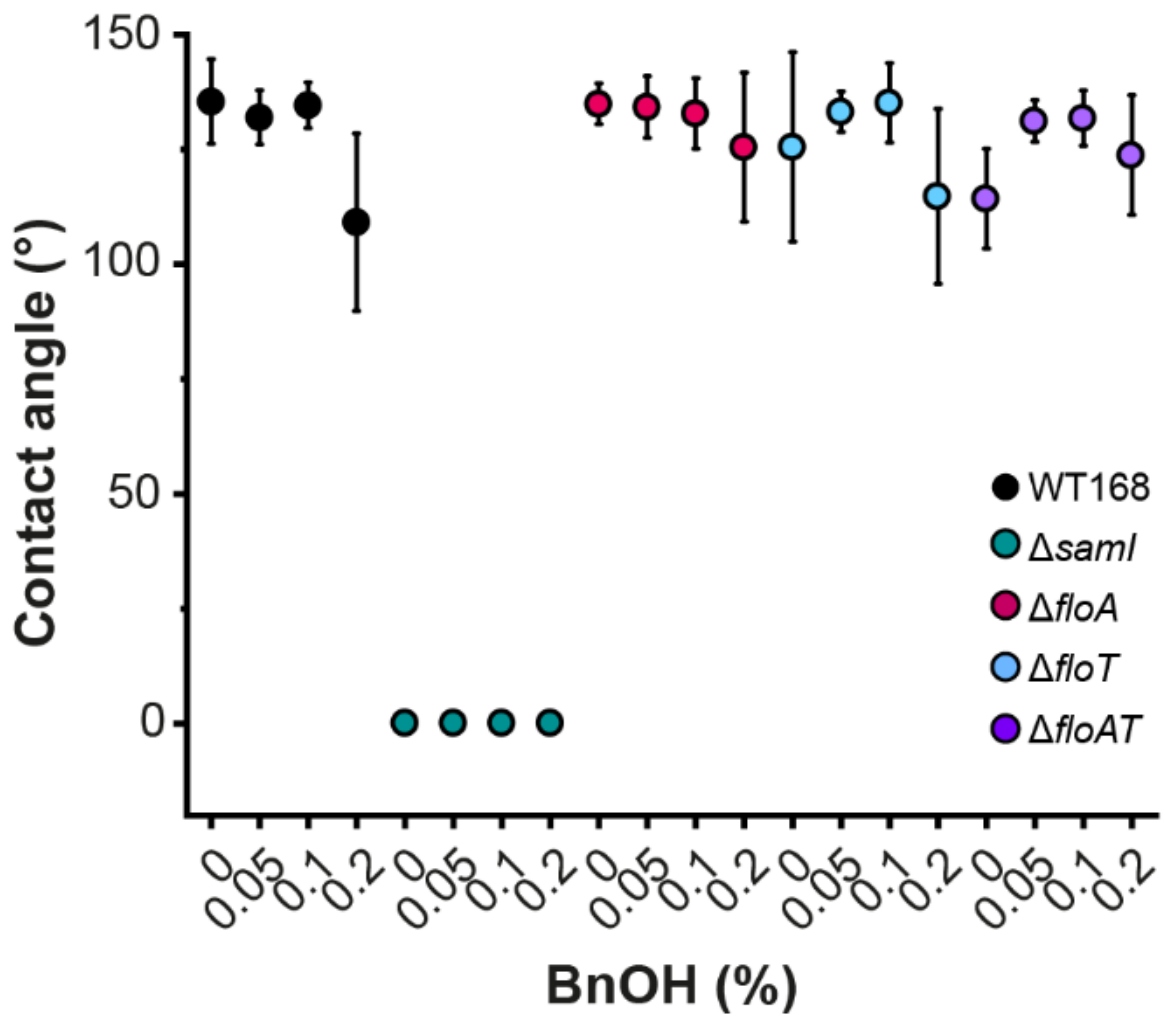

Supplementary Figure S5: Membrane fluidization does not improve Δ*samI's* biofilm phenotype. Surface hydrophobicity of SPFH mutants of LBGM supplemented with 0 % to 0.2 % benzyl alcohol. Contact angle and standard derivation were determined of three independent replicates.

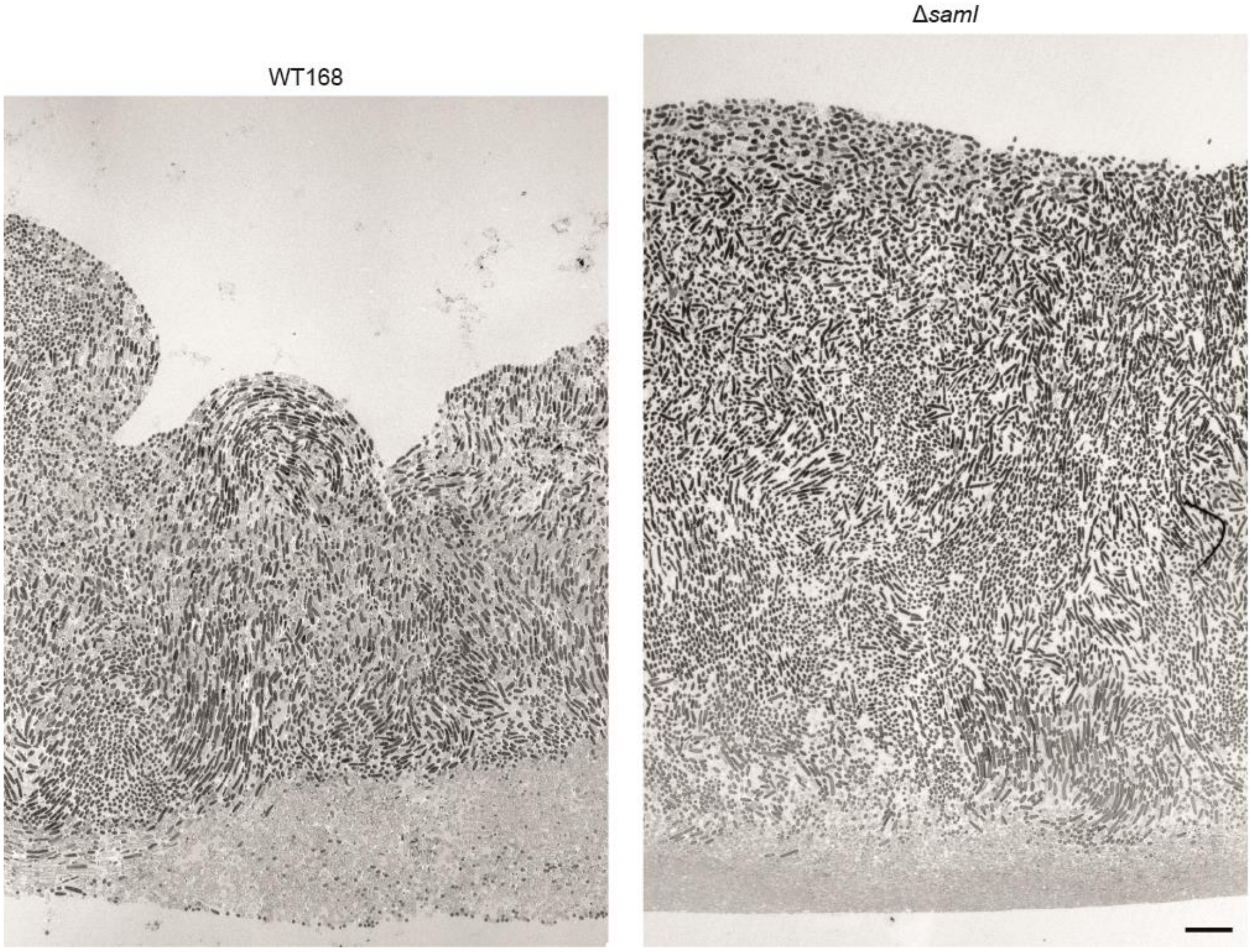

Supplementary Figure S6: Biofilms of WT168 and Δ*samI* have a layer of empty cells at the bottom of the biofilm. TEM images of showing an overview over cross section of WT168 (left) and Δ*samI* (right) biofilms. On the bottom of the biofilms a layer of empty cell envelopes is visible (scale: 10 µm).

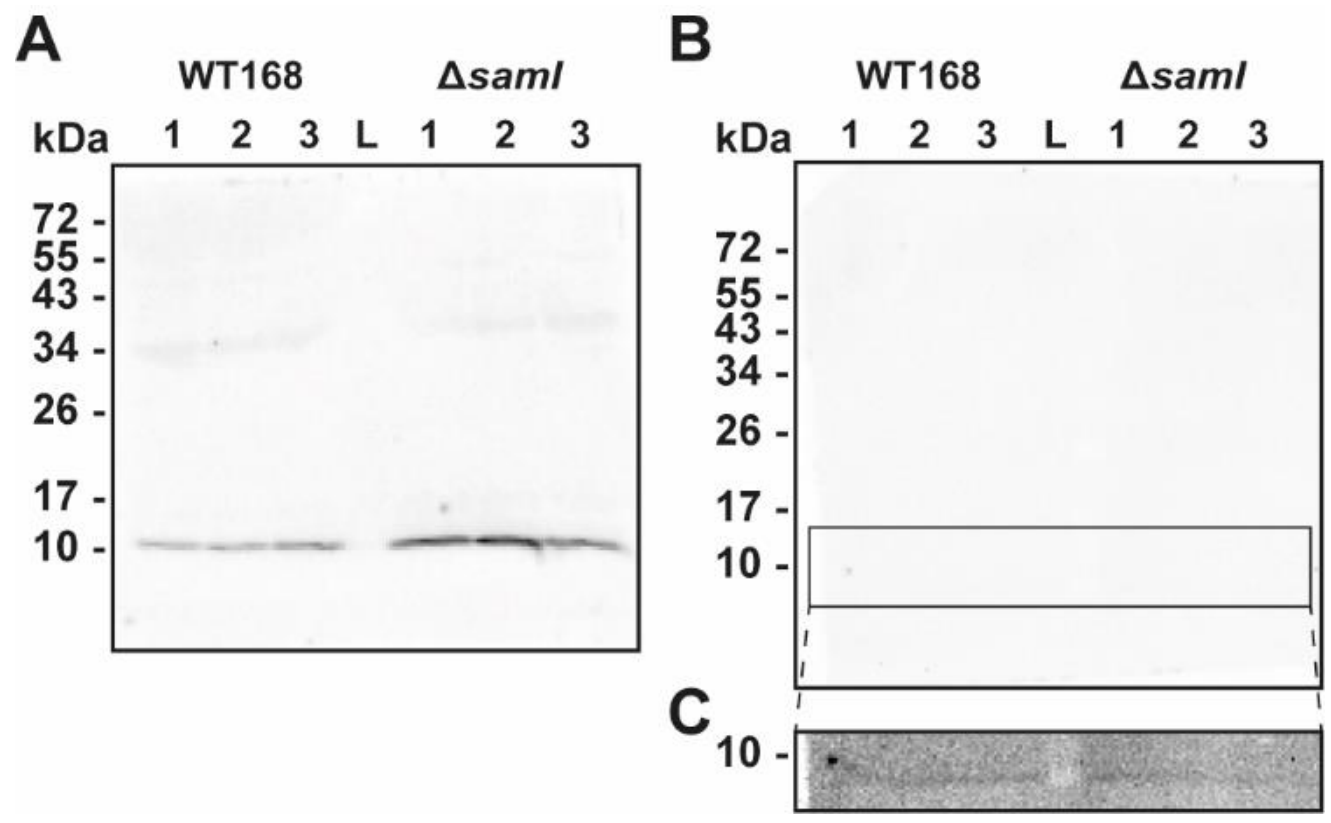

Supplementary Figure S7: AbrB levels in WT168 and Δ*samI* exponential cultures and biofilms. AbrB levels (10.6 kDa) were determined for three independent biological replicates in late exponential cultures (A) and of cells isolated from biofilms (B and C). The lane for the ladder is marked with L. For the Western Blot of biofilm samples (B) the area around 10 kDa was adjusted in brightness and contrast to enhance the very faint signal detected during western blotting (C).

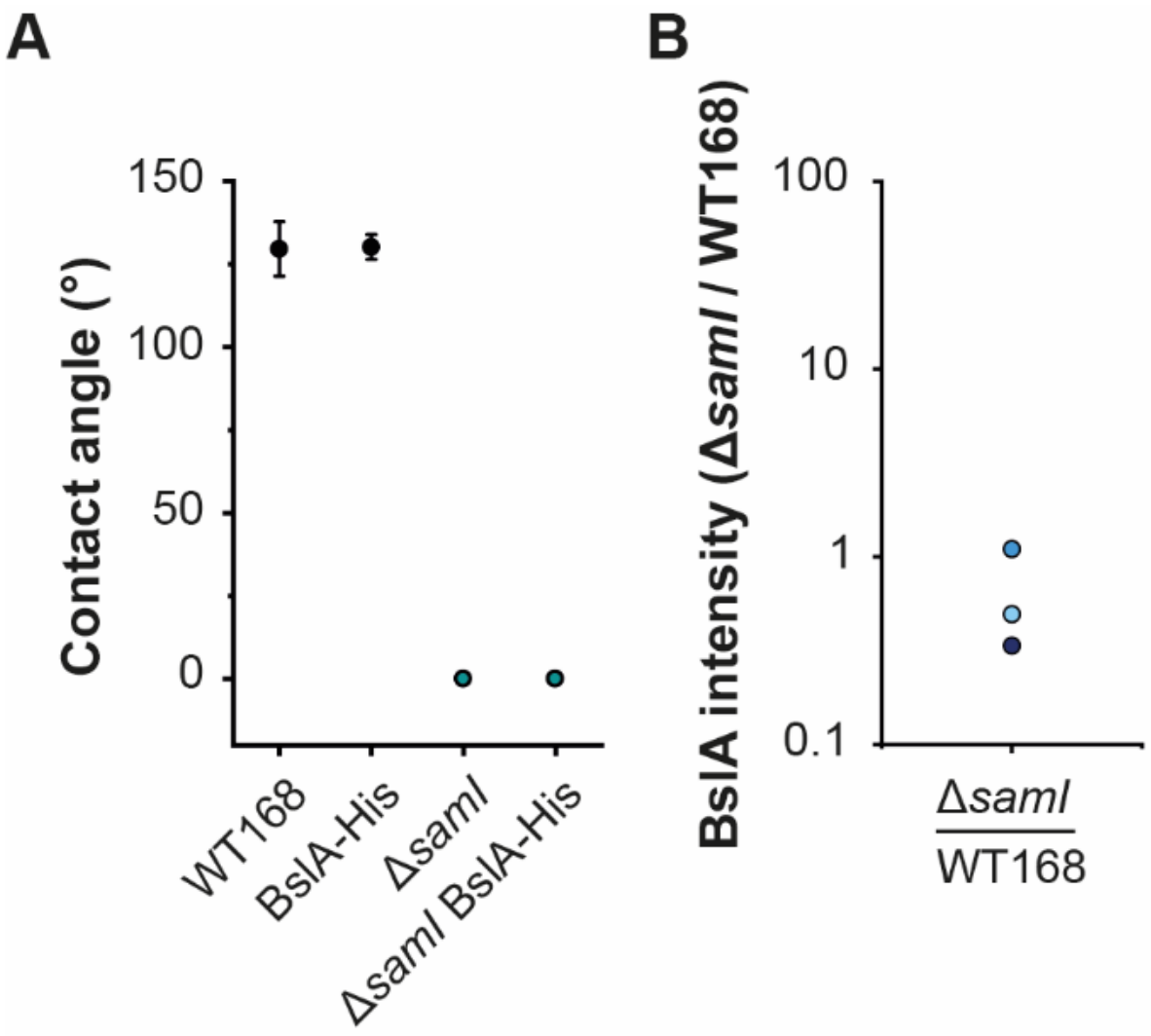

Supplementary Figure S8: BslA-His does not influence biofilm hydrophobicity and Δ*samI* exhibit reduced BslA-His levels. (A) Surface hydrophobicity of WT168 and Δ*samI* is not impacted by His tagged BslA. Contact angle and standard derivation were determined of three independent replicates. (B) Change in total BslA-His levels between WT168 and Δ*samI* isolated from biofilms. Three independent replicates are depicted in different colors.

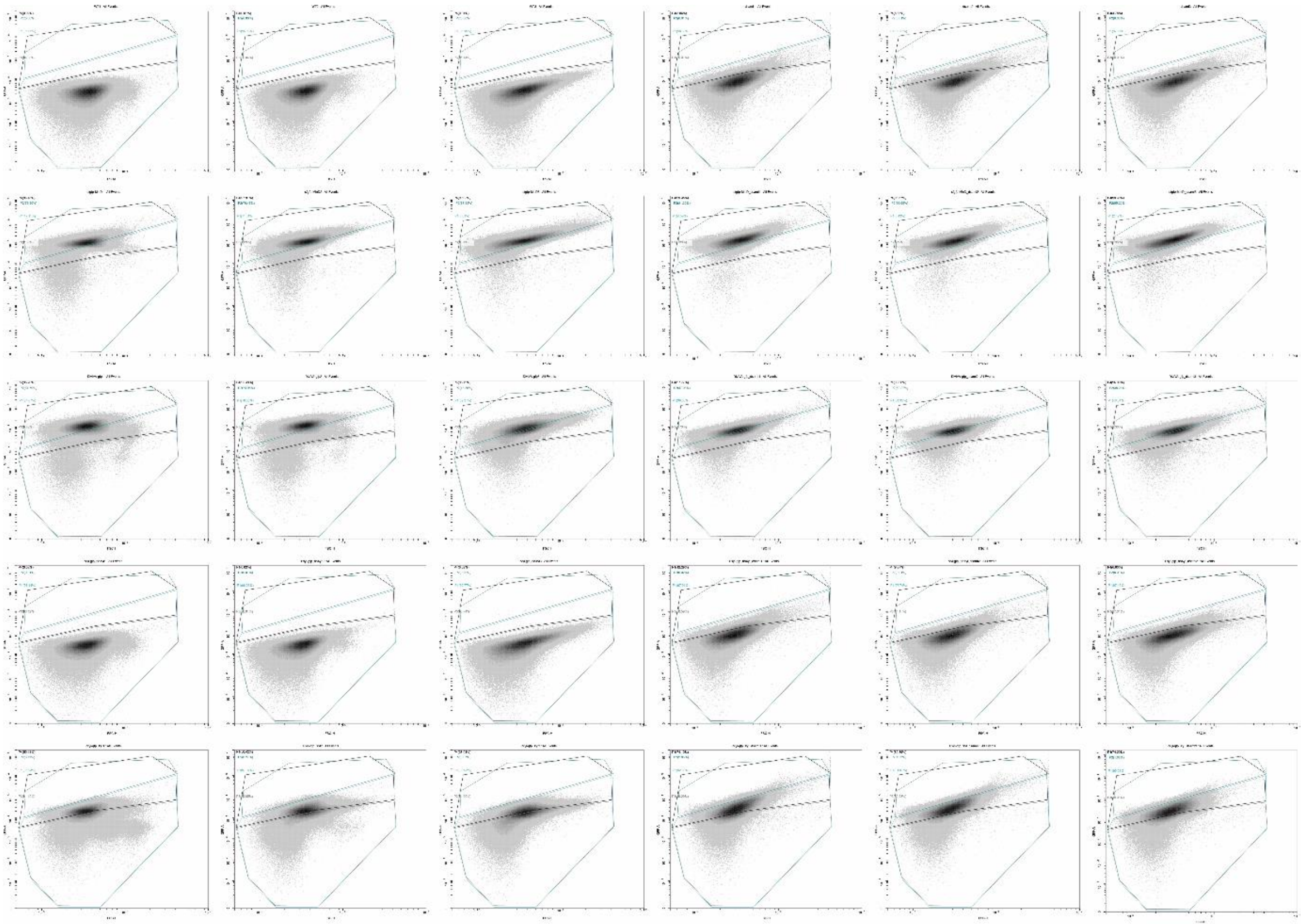

Supplementary Figure S9: Original blots of flow cytometric analysis of reporter strains exhibiting reduced abundance of fluorescent events in Δ*samI* background compared to WT168. WT168 and Δ*samI* were used as control to determine the placement of the gates (first row). P4 gate (black, corresponds to fluorescent population in WT168), P3 gate (gray, corresponds to non-fluorescent population in WT168), P2 gate (dark turquoise, corresponds to fluorescent population in Δ*samI*) and P1 gate (bright turquoise, corresponds to non-fluorescent population in Δ*samI*). msfGFP-MinD (second row), DivIVA-GFP (third row), P_{xyl}-GFP (0 % xylose, (fourth row), and GFP (1 % xylose, (fifth row)).

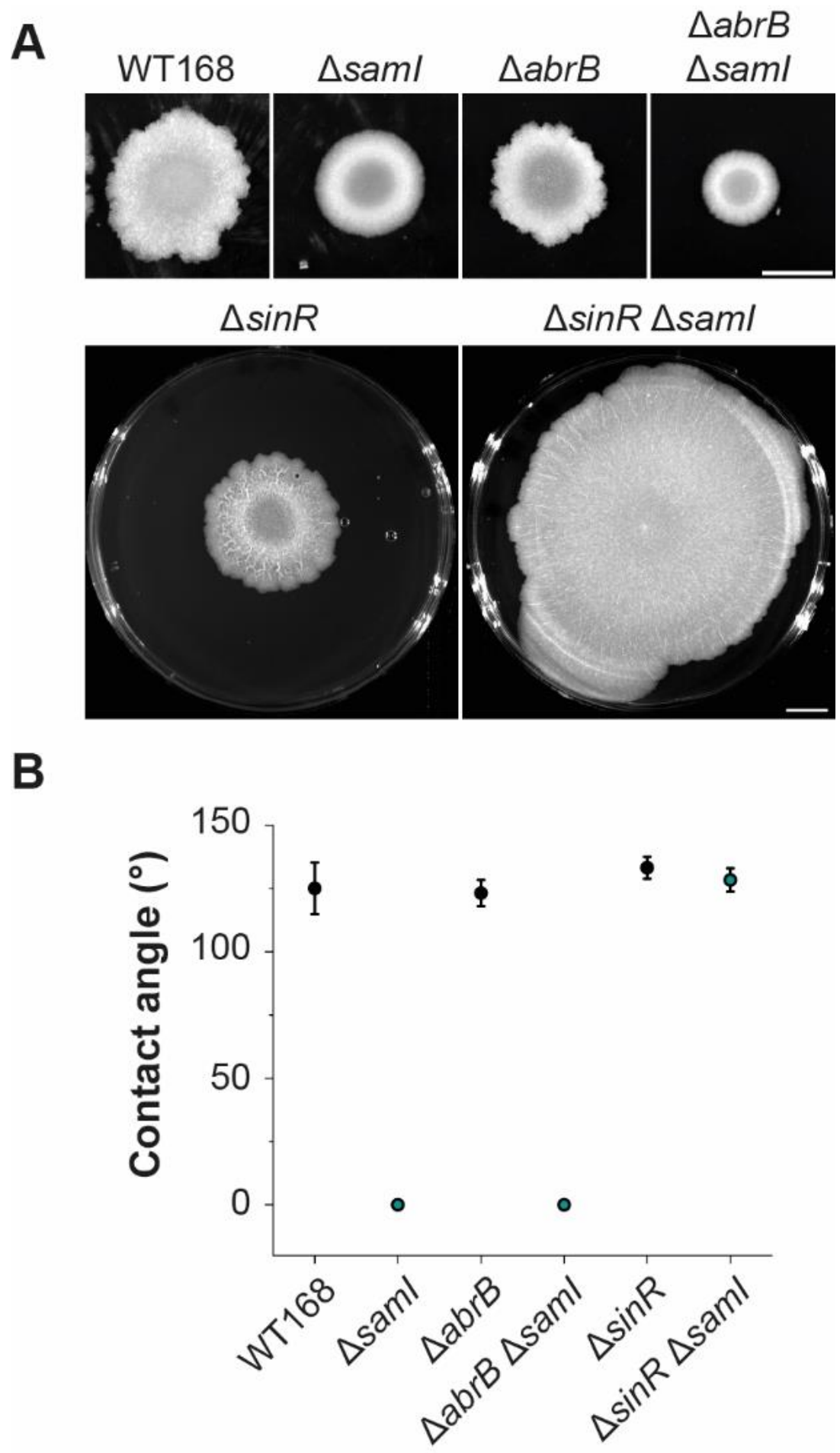

Supplementary Figure S10: Biofilm phenotype of Δ*samI* can be partially recovered upon additional deletion of Δ*sinR*. (A) Biofilm development of WT168 and Δ*samI* was determined upon additional deletion of Δ*abrB and* Δ*sinR* on LBGM agar (scale: 1 cm). (B) Surface hydrophobicity can be completely recovered upon additional loss of *sinR*. Average contact angle and standard derivation was determined in three independent replicates.

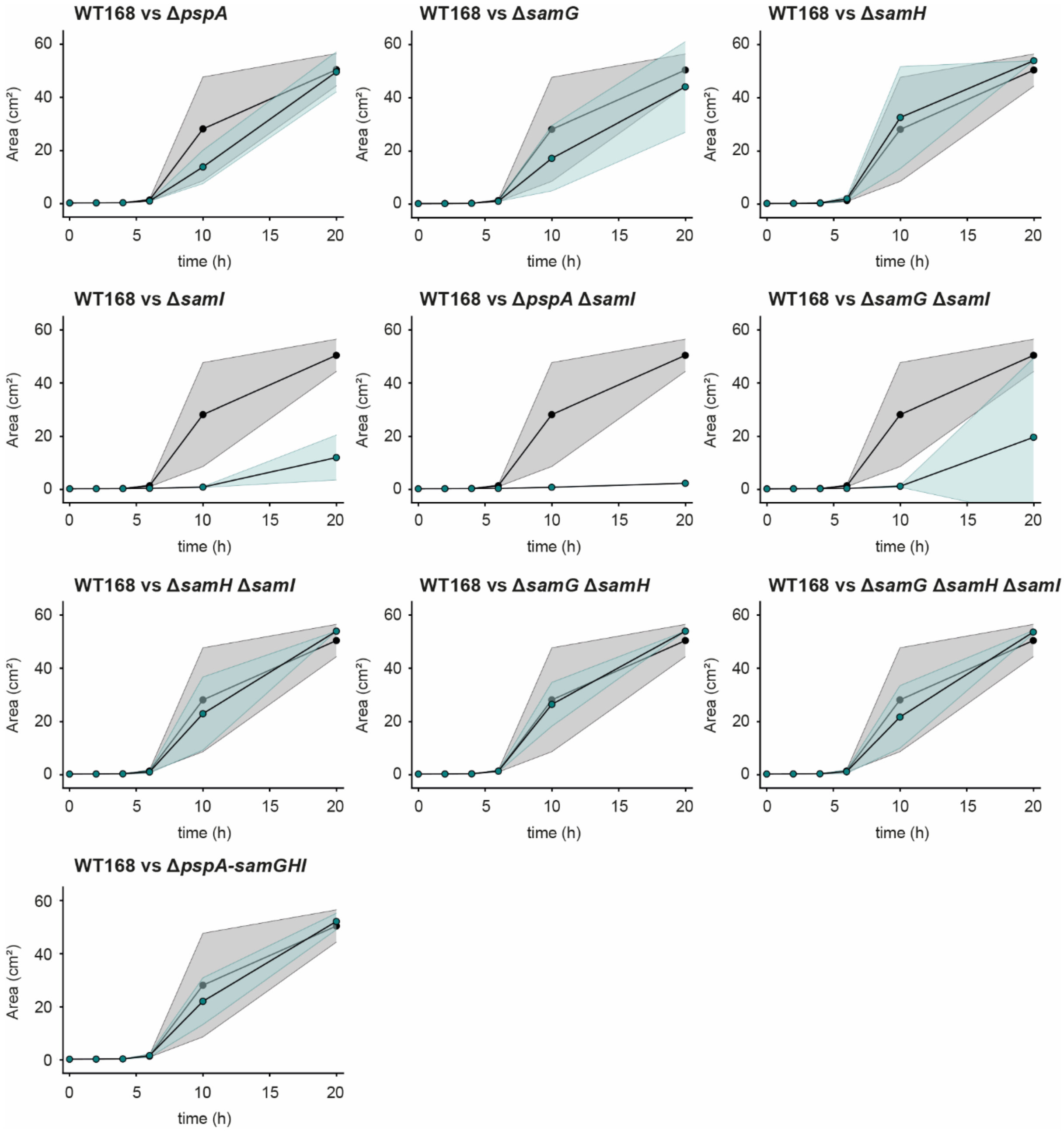

Supplementary Figure S11: Swimming progress of WT168, single, double, triple mutants, and the full operon knock-out of the *pspA-samGHI* operon. Swimming progress was observed over a time course of 20 h and average area (in cm²) and standard derivation were determined of three independent replicates.

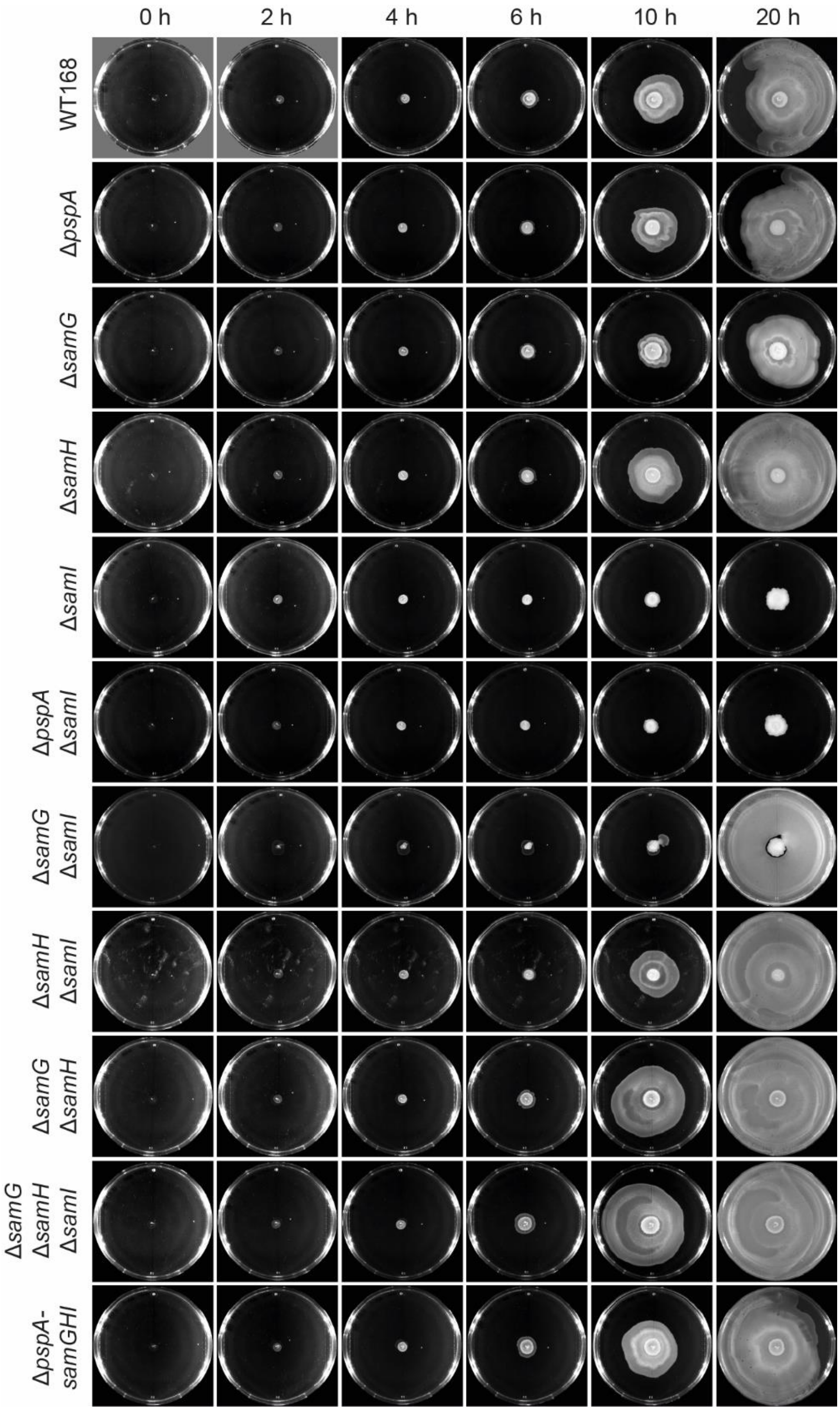

Supplementary Figure S12: Original images of swimming progress of one exemplary replicate. Swimming progress was determined for the WT168, single, double, triple mutants, and the full operon knock-out of the *pspA-samGHI* operon over a time course of 20 h. Swimming assays were performed in standard plates (diameter: 92 mm).

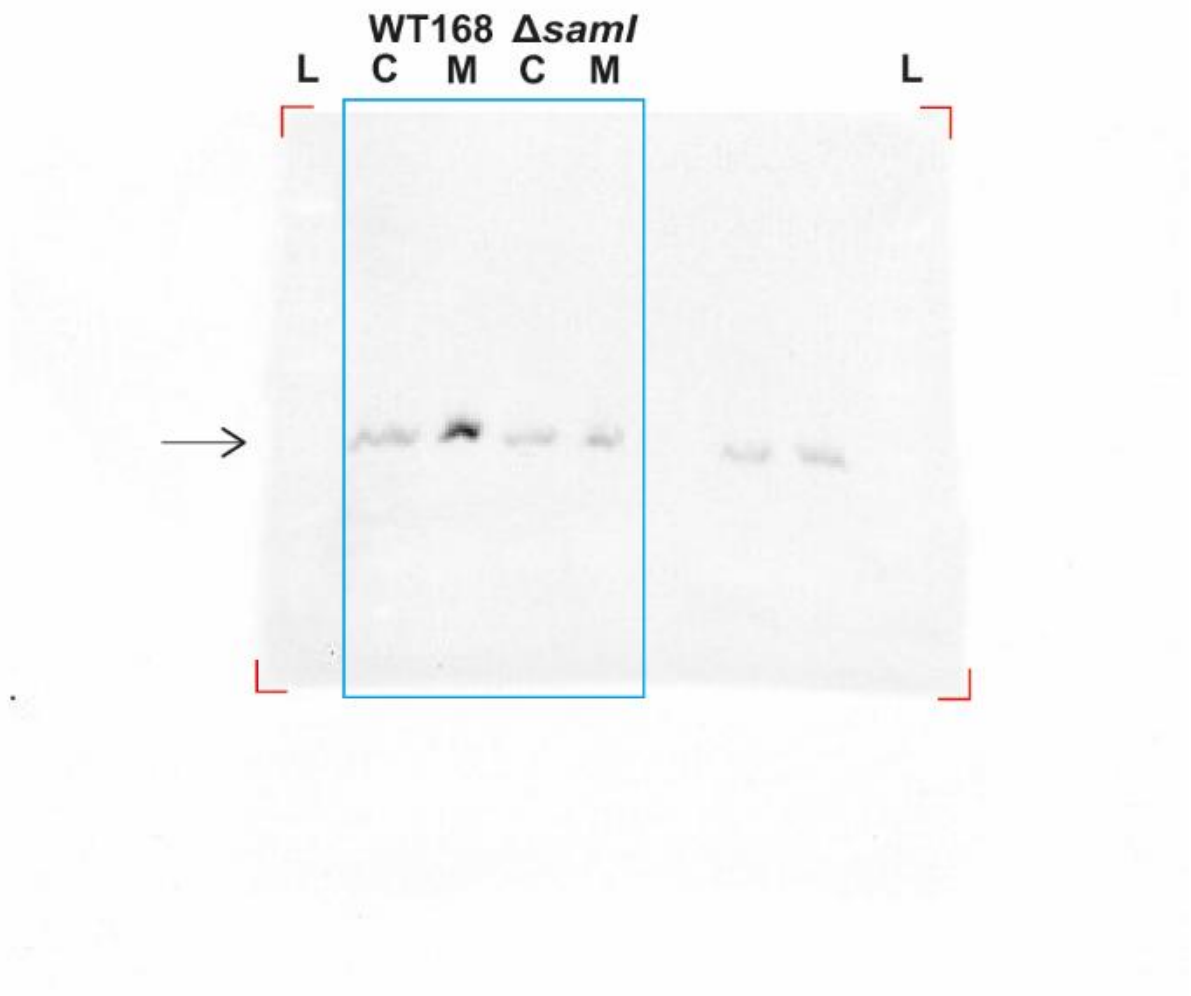

Supplementary Figure S13: Original unprocessed Western Blot 1, used for and shown in Figure 3C. Cellular (C) and matrix (M) fraction isolated from biofilms of WT168 and Δ*samI* were analyzed with Western Blotting for three independent replicates (first replicate shown here). The lanes for the ladder are marked with L. The corners of the Western Blot are marked with small red corners. Areas that were analyzed are inside the blue box. The BslA-His signal (20.2 kDa) is highlighted with an arrow. Western Blot 1 corresponds to TCE strained gel 1.

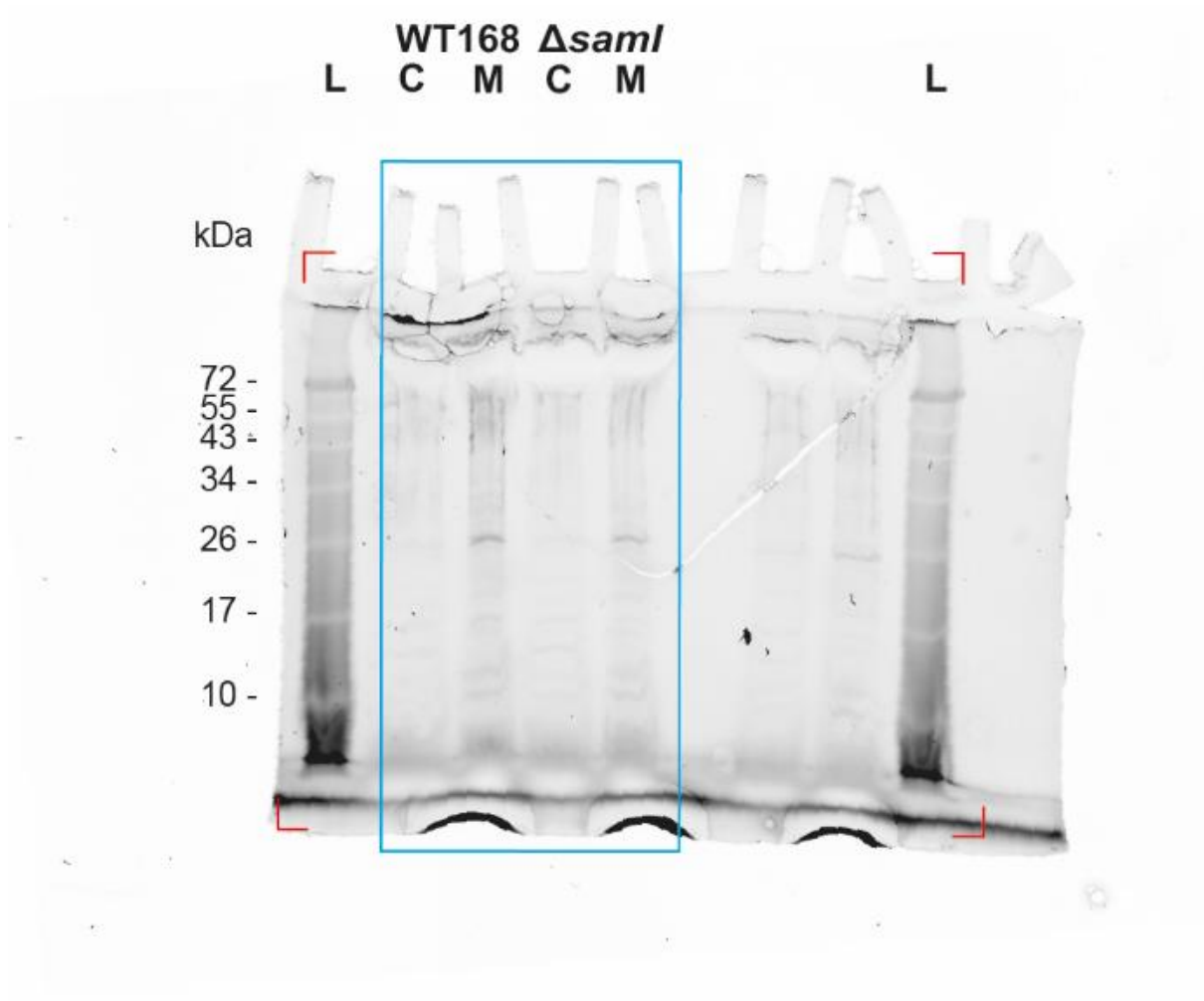

Supplementary Figure S14: Original unprocessed TCE stained gel 1, used for Figure 3C. Cellular (C) and matrix (M) fraction isolated from biofilms of WT168 and Δ*samI* were analyzed with Western Blotting for three independent replicates (TCE stained gel of first replicate shown here). The lane for the ladder are marked with L. For better visibility the annotation of the ladder is shown on the left side beside the gel. Areas of interest are inside the blue box. The corresponding localization of the corners of the Western Blot membrane have been visualized in the TCE stained gels with small red corners. TCE strained gel 1 corresponds to Western Blot 1.

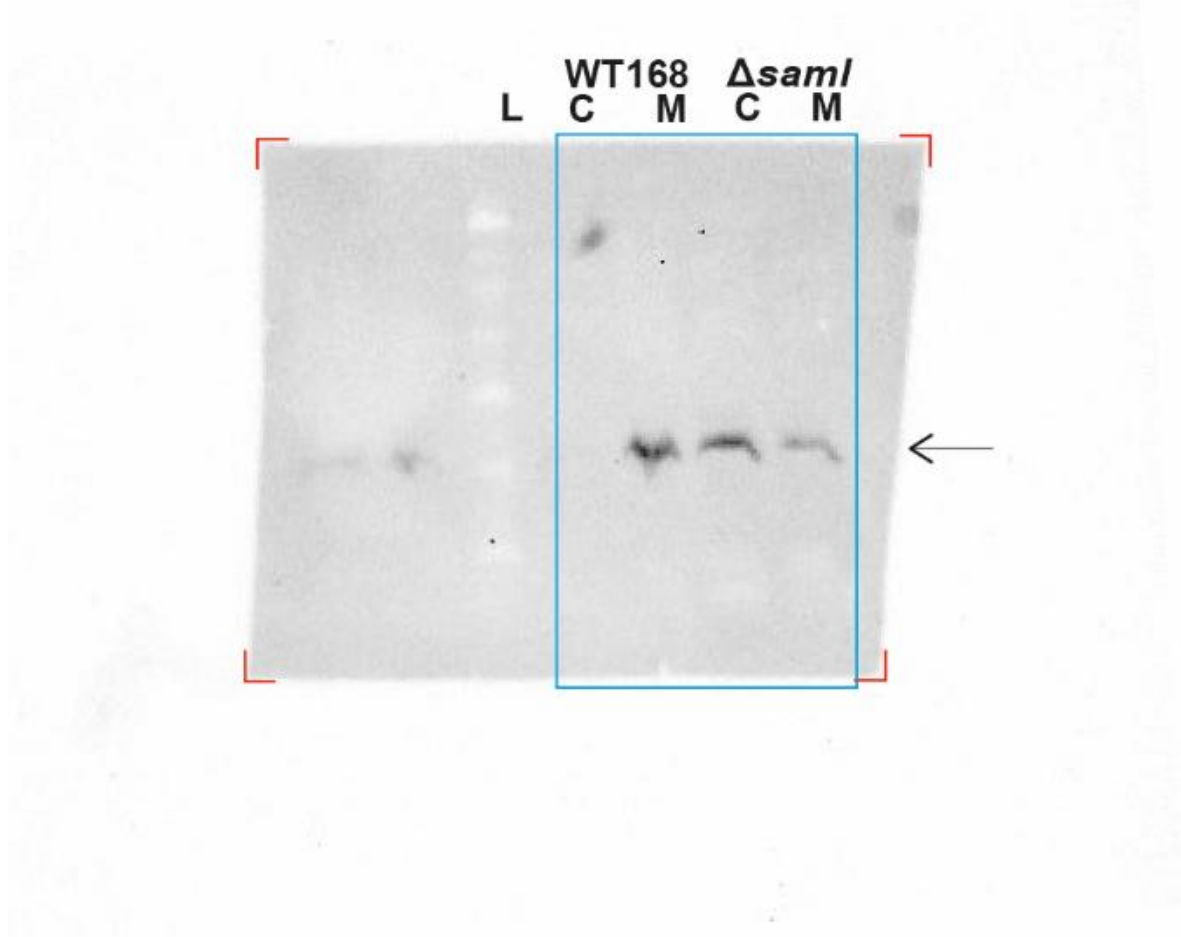

Supplementary Figure S15: Original unprocessed Western Blot 2, used for Figure 3C. Cellular (C) and matrix (M) fraction isolated from biofilms of WT168 and Δ*samI* were analyzed with Western Blotting for three independent replicates (second replicate shown here). The lane for the ladder is marked with L. The corners of the Western Blot are marked with small red corners. Areas that were analyzed are inside the blue box. The BslA-His signal (20.2 kDa) is highlighted with an arrow. Western Blot 2 corresponds to TCE strained gel 3.

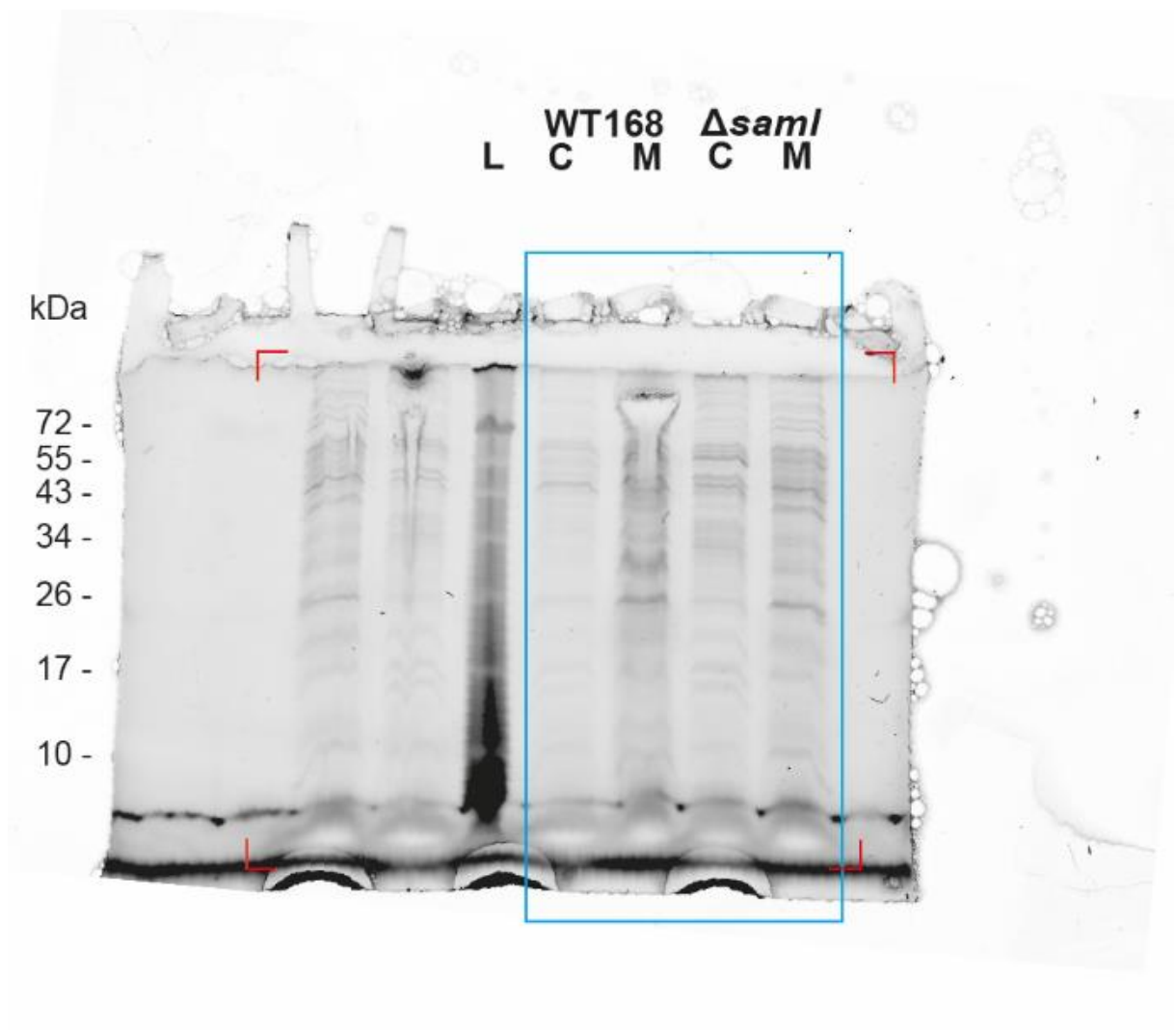

Supplementary Figure S16: Original unprocessed TCE stained gel 2, used for Figure 3C. Cellular (C) and matrix (M) fraction isolated from biofilms of WT168 and Δ*samI* were analyzed with Western Blotting for three independent replicates (TCE stained gel of second replicate shown here). The lane for the ladder is marked with L. For better visibility the annotation of the ladder is shown on the left side beside the gel. Areas of interest are inside the blue box. The corresponding localization of the corners of the Western Blot membrane have been visualized in the TCE stained gels with small red corners. TCE strained gel 2 corresponds to Western Blot 2.

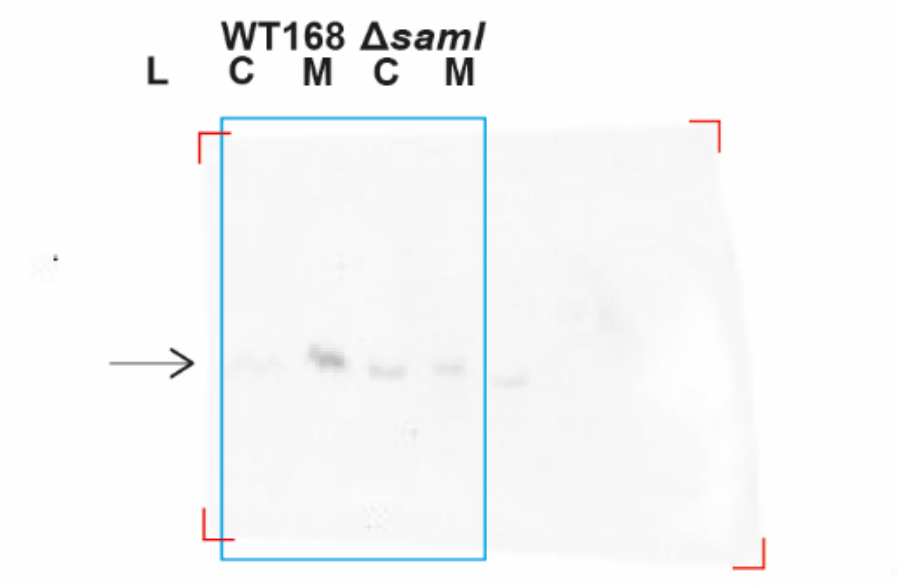

Supplementary Figure S17: Original unprocessed Western Blot 3, used for Figure 3C. Cellular (C) and matrix (M) fraction isolated from biofilms of WT168 and Δ*samI* were analyzed with Western Blotting for three independent replicates (third replicate shown here). The lane for the ladder is marked with L. The corners of the Western Blot are marked with small red corners. Areas that were analyzed are inside the blue box. The BslA-His signal (20.2 kDa) is highlighted with an arrow. Western Blot 3 corresponds to TCE strained gel 3.

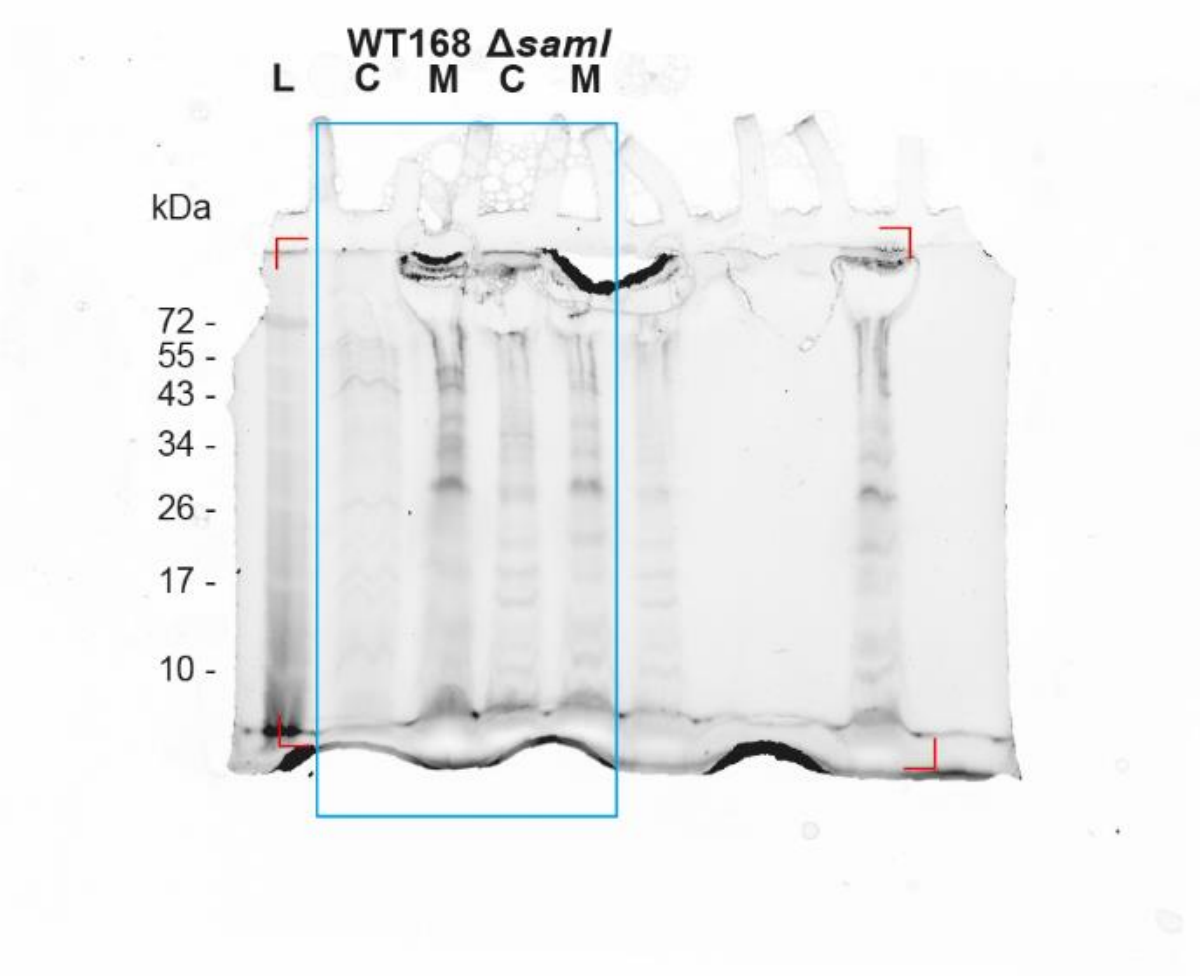

Supplementary Figure S18: Original unprocessed TCE stained gel 3, used for Figure 3C. Cellular (C) and matrix (M) fraction isolated from biofilms of WT168 and Δ*samI* were analyzed with Western Blotting for three independent replicates (TCE stained gel of third replicate shown here). The lane for the ladder is marked with L. For better visibility the annotation of the ladder is shown on the left side beside the gel. Areas of interest are inside the blue box. The corresponding localization of the corners of the Western Blot membrane have been visualized in the TCE stained gels with small red corners. TCE strained gel 3 corresponds to Western Blot 3.

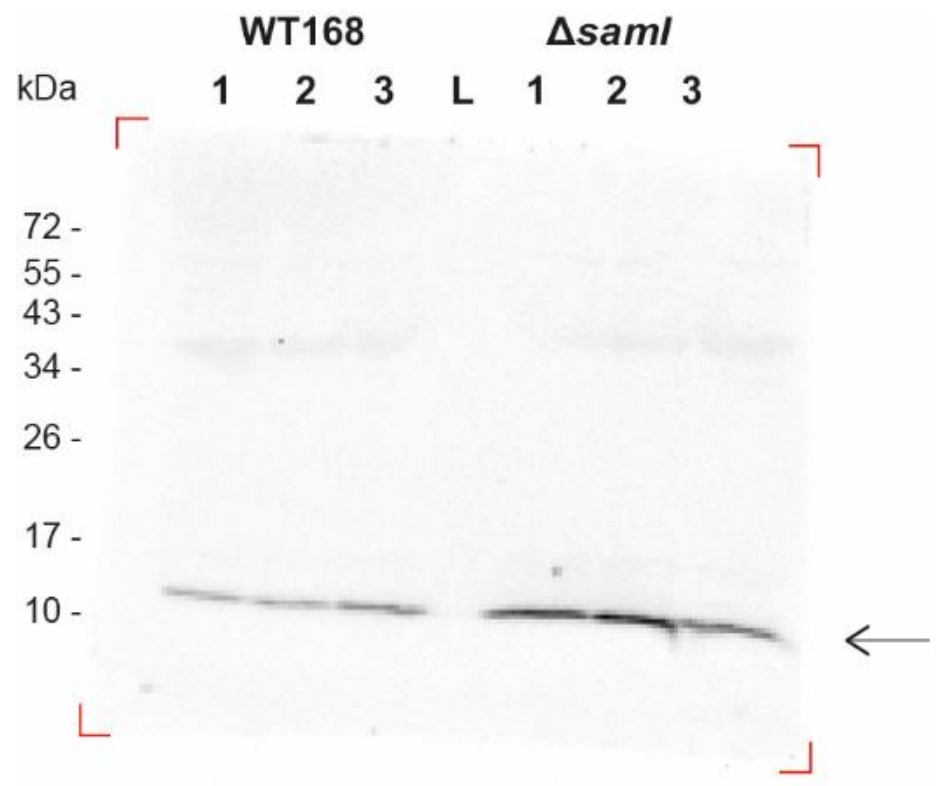

Supplementary Figure S19: Original unprocessed Western Blot 4, used for Figure S8A. Three independent replicates (1 to 3) of exponentially growing WT168 and Δ*samI* cultures were analyzed with Western Blotting. The lane for the ladder is marked with L. The corners of the Western Blot are marked with small red corners. The AbrB signal (10.6 kDa) is highlighted with an arrow. Western Blot 4 corresponds to TCE strained gel 4.

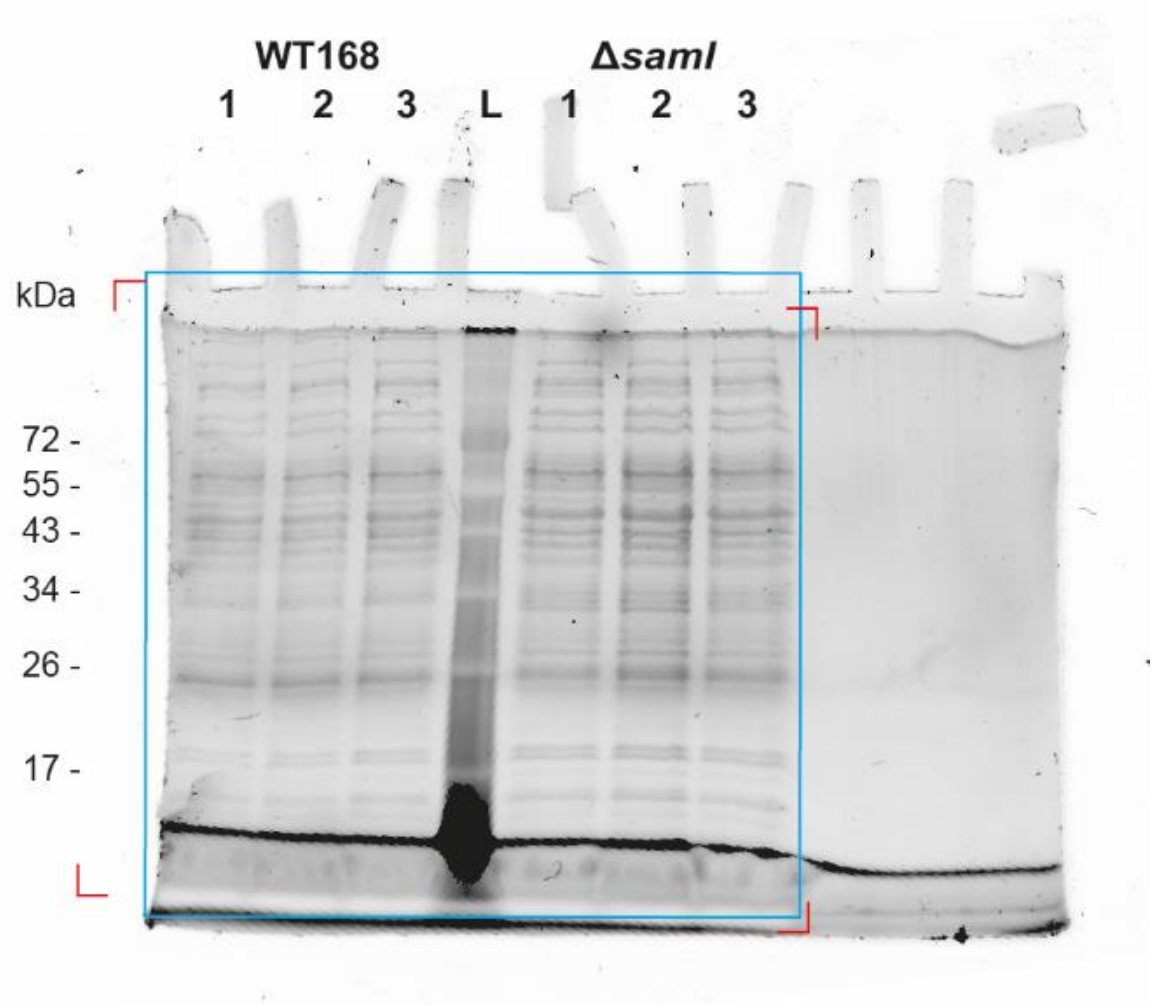

Supplementary Figure S20: Original unprocessed TCE stained gel 4, used for Figure S8A. Three independent replicates (1 to 3) of exponentially growing WT168 and Δ*samI* cultures were analyzed with Western Blotting (TCE stained gel of western blot 4 shown here). The lane for the ladder is marked with L. For better visibility the annotation of the ladder is shown on the left side beside the gel. The corresponding localization of the corners of the Western Blot membrane have been visualized in the TCE stained gels with small red corners. TCE strained gel 4 corresponds to Western Blot 4.

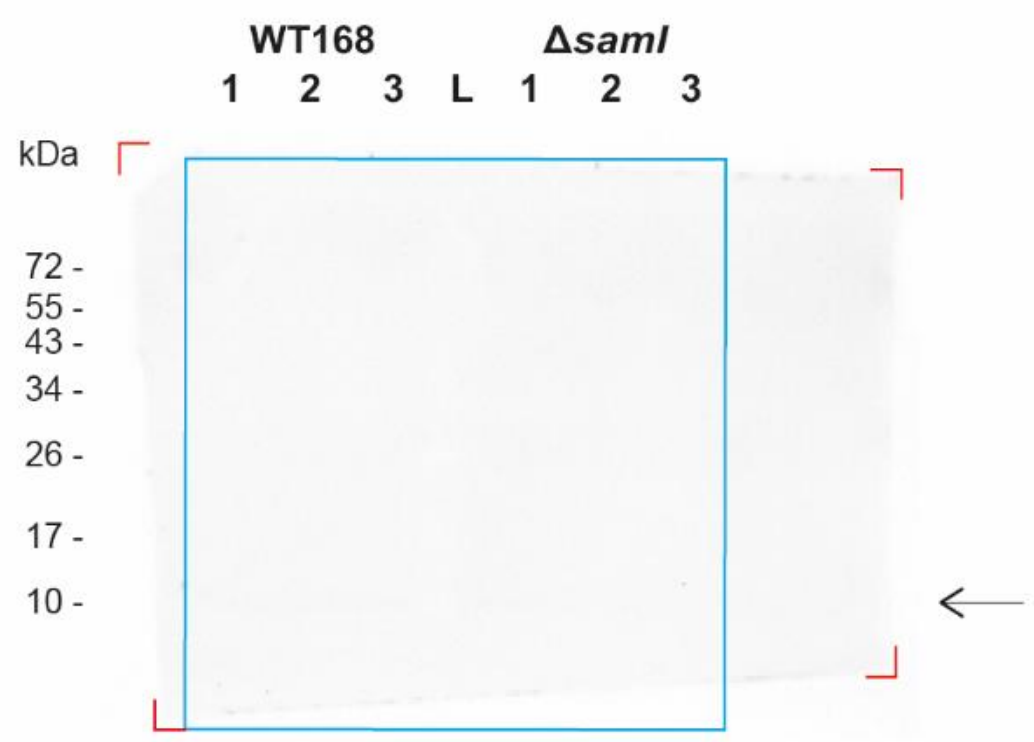

Supplementary Figure S21: Original unprocessed Western Blot 5, used for Figure S8B and C. Three independent replicates (1 to 3) of WT168 and Δ*samI* biofilms were analyzed with Western Blotting. The lane for the ladder is marked with L. The corners of the Western Blot are marked with small red corners. The region where AbrB signal (10.6 kDa) was detected upon editing (see figure S8 for more information) is highlighted with an arrow. Western Blot 5 corresponds to TCE strained gel 5.

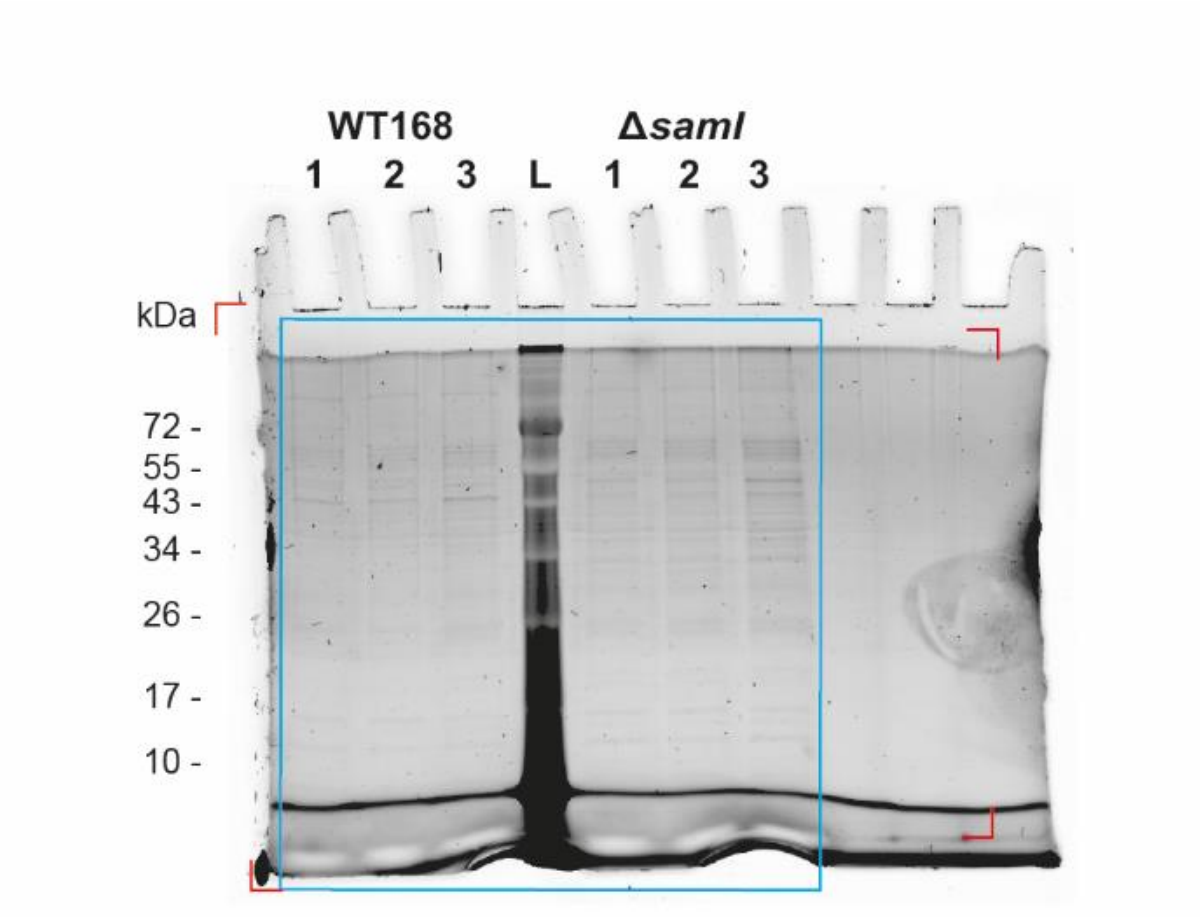

Supplementary Figure S22: Original unprocessed TCE stained gel 5, used for Figure S8B and C. Three independent replicates (1 to 3) of WT168 and Δ*samI* biofilms were analyzed with Western Blotting (TCE stained gel of western blot 5 shown here). The lane for the ladder is marked with L. For better visibility the annotation of the ladder is shown on the left side beside the gel. The corresponding localization of the corners of the Western Blot membrane have been visualized in the TCE stained gels with small red corners. TCE strained gel 5 corresponds to Western Blot 5.

2. Supplementary tables

Table S1: ***Bacillus subtilis*** **strains used in this study.**

Strain number	Name	Genotype/description	Reference
SBB001	WT168	*trpC2*	Laboratory collection, derived from D. Scheffers lab
SBB002	WT168 Δ*floT*	*trpC2 yuaG::pMUTIN4 erm*	This work
SBB003	WT168 Δ*samI*	*trpC2 samI::kan*	This work
SBB004	WT168 Δ*pspA*	*trpC2 pspA::kan*	This work
SBB005	WT168 Δ*samH*	*trpC2 samH::kan*	This work
SBB006	WT168 Δ*samG*	*trpC2 samG::kan*	This work
SBB007	WT168 Δ*pspA-samGHI*	*trpC2, pspA-samGHI::(*P_{pspA} *erm)*	This work
SBB008	NCIB 3610 (DSM 10, ATCC 6051)	Undomesticated wild type strain	Laboratory collection
SBB009	NCIB 3610 Δ*samI*	*samI::kan*	This work
SBB041	WT168 Δ*ytrF*	*trpC2 ytrF::kan*	This work
SBB060	WT168 Δ*samI* P_{hs}-*samI*	*trpC2 samI::kan amyE::*$P_{hyperspank}$-*samI spec*	This work
SBB066	WT168 Δ*bslA*	*trpC2 bslA::kan*	This work
SBB067	WT168 Δ*samG* Δ*samI* P_{hs}-*samI*	*trpC2 samG::erm samI::kan amyE::*$P_{hyperspank}$-*samI spec*	This work
SBB070	WT168 Δ*samG*	*trpC2 samG::erm*	This work
SBB071	WT168 Δ*floA*	*trpC2 yqfA::tet*	This work
SBB094	WT168 Δ*samI*	*trpC2 ΔsamI*	This work
SBB096	WT168 Δ*abrB*	*trpC2 abrB::kan*	This work
SBB100	WT168 Δ*floT* Δ*floA*	*trpC2 yuaG::pMUTIN4 erm yqfA::tet*	This work
SBB102	WT168, P_{yxl}-GFP	*trpC2, amyE::Pyxl-gfpmut1 spec*	This work
SBB127	WT168 BslA-His	*trpC2 bslA::bslA-6xHis-cat*	This work
SBB129	WT168 Δ*samI* BslA-His	*trpC2 samI::kan bslA::bslA-6xHis-cat*	This work
SBB131	WT168 Δ*ytrF* BslA-His	*trpC2 ytrF::kan bslA::bslA-6xHis-cat*	This work
SBB133	WT168 DivIVA-GFP	*trpC2 divIVA::divIVA-gfp cat*	This work
SBB134	WT168 Δ*samI* DivIVA-GFP	*trpC2 samI::kan divIVA::divIVA-gfp cat*	This work
SBB136	WT168 Δ*samI* Δ*pspA*	trpC2 Δ*samI pspA::kan*	This work
SBB137	WT168 Δ*samI* Δ*samG*	trpC2 Δ*samI samG::kan*	This work
SBB138	WT168 Δ*samHI*	*trpC2 samH-samI::erm*	This work
DS8521	WT168 Δ*fliM*, $P_{fla/che}$ FliM-GFP	*trpC2 ΔfliM amyE::*$P_{fla/che}$-*fliM–GFP spec*	This work
SBB149	WT168 Δ*fliM*	*trpC2 fliM::kanR*	This work
SBB150	WT168 Δ*samI* Δ*fliM*	*trpC2 ΔsamI fliM::kanR*	This work
SBB151	WT168 Δ*fliM* $P_{fla/che}$ FliM-GFP	*trpC2 fliM::kanR amyE::*$P_{fla/che}$-*fliM–GFP spec*	This work

SBB152	WT168 Δ*samI* Δ*fliM* $P_{fla/che}$ FliM-GFP	*trpC2 ΔsamI fliM::kanR amyE::*$P_{fla/che}$*-fliM–GFP spec*	This work
SBB159	WT168 Δ*samGH*	*trpC2 samG-samH::erm*	This work
SBB160	WT168 Δ*samGHI*	*trpC2 samG-samH-samI::erm*	This work
SBB193	WT168 Δ*samI* P_{hs}-*samH*	*trpC2 samI::kan amyE::*$P_{hyperspank}$*-samH spec*	This work
SBB241	WT168 Δ*samI* Δ*abrB*	*trpC2 ΔsamI abrB::kan*	This work
SBB243	WT168 Δ*sinR*	*trpC2 sinR::kan*	This work
SBB244	WT168 Δ*samI* Δ*sinR*	*trpC2 ΔsamI sinR::kan*	This work
SBB245	WT168 Δ*samI*, P_{yxl}-GFP	*trpC2 samI::kan, amyE::Pyxl-gfpmut1 spec*	This work
SBB246	WT168 msfGFP-MinD	*trpC2 minD::aad9-msfGFP-minD*	This work
SBB247	WT168 Δ*samI* msfGFP-MinD	*trpC2 samI::kan minD::aad9-msfGFP-minD*	This work
DB003	WT168 Δ*floT*	*trpC2 yuaG::pMUTIN4 erm*	(Donovan & Bramkamp, 2009)
BB001	WT168 Δ*floA*	*trpC2 yqfA::tet*	(Bach & Bramkamp, 2013)
1803	DivIVA-GFP	*divIVA::(*$P_{divIVA\text{-}gfp}$ *divIVA* $^{+}$ *cat)*	(Edwards *et al.*, 2000)
DS8521	WT168 Δ*fliM,* $P_{fla/che}$ FliM-GFP	*ΔfliM amyE::*$P_{fla/che}$*-fliM–GFP spec*	(Guttenplan *et al.*, 2013)
	WT168 Δ*pspA-samGHI*	*trpC2, pspA-samGHI::(*P_{pspA} *erm)*	Gift from T. Mascher

Table S2: *Escherichia coli* strains used in this study.

Strain number	Name	Reference
SBE002	DH5α	Laboratory collection
SBE005	DH5α pKill	Laboratory collection
SBE011	DH5α pDR111-*samI*	This work
SBE013	DH5α pDR244	(Koo *et al.*, 2017)
SBE014	DH5α pSG1729	(Lewis & Marston, 1999)
SBE023	DH5α pKill-*samI-halo-spec*	A. Lassota
SBE024	DH5α pKill-*spec-halo-samI*	A. Lassota
SBE029	DH5α pKill-Δ*samHI*	This work
SBE030	DH5α pKill-Δ*samGH*	This work
SBE031	DH5α pKill-Δ*samGHI*	This work
SBE041	DH5α pDR111-*samH*	This work
	XL Blue1 pDRyuaB2	(Kovács & Kuipers, 2011)

Table S3: Plasmids used in this study.

Name	Description	References
pKill	pUC18mut with *txpA* (*yqdB*) gene	Laboratory collection
pDR244	temperature-sensitive plasmid with constitutively expressed Cre recombinase	(Koo *et al.*, 2017)
pDR111-*samI*	For ectopic expression of *samI* under the $P_{hyperspank}$ promoter (IPTG inducible)	This work

pKill-Δ*samHI*	For generation of a double deletion of *samH* and *samI*	This work
pKill-Δ*samGH*	For generation of a double deletion of *samG* and *samH*	This work
pKill-Δ*samGHI*	For generation of a triple deletion of *samG*, *samH*, and *samI*	This work
pDR111-*samH*	For ectopic expression of *samH* under the $P_{hyperspank}$ promoter (IPTG inducible)	This work
pKill-*samI-halo-spec*	C-terminal *samI-halo* fusion	A. Lassota
pKill-*spec-halo-samI*	N-terminal *samI-halo* fusion	A. Lassota
pDRyuaB2	For ectopic expression of *bslA* under the $P_{hyperspank}$ promoter (IPTG inducible)	(Kovács & Kuipers, 2011)
pHF2	pUC18mut-*minDup-aad9-msfGFP-minD*	(Feddersen *et al.*, 2021)
pSG1729	*gfpmut1* under xylose-inducible promoter, for integration into *amyE* locus	(Lewis & Marston, 1999)

Table S4: Oligonucleotides used in this study.

Primer Number	Primer	Sequence 5'→ 3'
SB001	samI_500bp_upstream_fw	ACGACCAAAAGCACAAAC
SB027	pDR111_fwd	AAGCTTAATTGTTATCCGCT
SB028	pDR111_rev	GCATGCAAGCTAATTCGGTG
SB029	samI_pDR111_fwd	CACCGAATTAGCTTGCATGCTTATACAAGCTTCTGGCC
SB030	samI_pDR111_rev	AGCGGATAACAATTAAGCTTAAAGGGAGAGGCGTAATG
SB035	pDR111_seq_fw	GGCAAGAACGTTGCTCGAG
SB036	pDR111_seq_rv	TCAGCCGACTCAAACATCAA
SB037	pKill_fwd	GCTTGGCACTGGCCGTCGTTT
SB038	pKill_rev	TTGCATGCCTGCAGGTCGACT
SB052	samI_500bp_downstream_rv	CCCCATCCTTCATTTTTACCA
SB080	divIVA_fw	GAGCTTGAAGCGAAAGTCAATGAG
SB081	divIVA_rv	TCGTTGATAATGCGATCAGCG
SB086	spo0A_fw	TGGGCAGGAAGATGTCACG
SB087	spo0A_rv	CACGATGCGTCACACTGCTG
SB088	abrB_fw	CCTATCGAACTGCGTCGTACTC
SB089	abrB_rv	CCGCCTGCAAGTTTAAGGTTATC
SB090	lip_fw	CAATCCAGTCGTTATGGTTCAC
SB091	lip_rv	CGTGATAATACCGGTCCATTG
SB092	degQ_fw	CAATTGTTATTCCGACTCGAACTTG
SB093	degQ_rv	AATTGTATTTATCGAGTTGATCAATGC
SB098	tasA_fw	CCGGGAGATAAGTTGACAAAGG
SB099	tasA_rv	GTAGCCATTGCCGCCCTCTT
SB100	bslA_fw2	GTTACTCGTTTCTGCACC
SB101	bslA_rv2	GGACGGTAAGTCAATTCG
SB102	sinR_fw	GTCTCCGCTGTTCTGGACG
SB103	sinR_rv	CGGATGTCATCGCATCGC

SB104	bslA_fwd	CCTCTAGAGTCGACCTGCAGGCATGCAAATGAAACGCAAATTATTATCTTCTTTGGC
SB105	bslA_GG_6xHis_rev	ATTTTTATCTAAAGATGATGATGATGATGATGCCCTCCGTTGCAACCGCAAGGCTGAG
SB106	catR_GG_6xHis_fwd	CTTGCGGTTGCAACGGAGGGCATCATCATCATCATCATCTTTAGATAAAAATTTAGGAGGCATATC
SB111	catR_rev	ACCGGTCTTTTACATTATAAAAGCCAGTCATTAGGC
SB114	bslA_dw_fwd_2	ATAATGTAAAAGACCGGTTAACGCC
SB115	bslA_dw_rev_2	GTTGTAAAACGACGGCCAGTGCCAAGCCGATGGCAACAATCACATATTC
SB116	samI_fw	GCCAAGGGAATTCAAGAGGACTTA
SB117	samI_rv	CCAACCATACCGTAGGAAGCG
SB118	pKill_seq_fw	GGATCCTCTAGAGTCGACC
SB119	pKill_seq_rv	CTGCAAGGCGATTAAGTTG
SB137	up_ldh	TACCCTGGAAGGATGATTA
SB138	dw_ldh	TACTCTAAAGTTGCGGTTAG
SB160	samI-pKill_GH(I)_rv	ATAAAGCATTTTGCATGCCTGCAGGTCGAC
SB161	pspA_GH(I)_fw	AGGCATGCAAAATGCTTTATTGGACAAGGC
SB162	pspA_GH(I)_rv	TCTCGCCTGCTTACTTATCGAGCATCATTTTCG
SB163	ermR_GH(I)_fw	CGATAAGTAAGCAGGCGAGAAAGGAGAGAG
SB164	ermR_GH_rv	AAAACGACATCGAGGCTCCTGTCACTGCTTC
SB165	samI-dw-pKill_(G)HI_fw	GAATCATACAGTGAAAAGTCCGGAG
SB166	ermR_(G)HI_rv	GACTTTTCACTGTATGATTCCGAGGCTCCTGTCACTGCTTC
SB167	samI-dw-pKill_HI_rv	CAGTTTGCTCTTGCATGCCTGCAGGTCGAC
SB168	samG_HI_fw	AGGCATGCAAGAGCAAACTGTACCAGAGTCC
SB169	samG_HI_rv	TCTCGCCTGCTCAAAATCCGCCTCCCATCATC
SB170	ermR_HI_fw	CGGATTTTGAGCAGGCGAGAAAGGAGAGAG
SB257	pDR111_rev	TACGCCTCTCCCTTTAAGCTTA
SB258	samH_fwd	AGCTTAAAGGGAGAGGCGTAATGCGTGGATTTTTTGGG
SB259	samH/GH_rev	CACCGAATTAGCTTGCATGCTTAAAAACTGCCCCGGCTC

Table S5: Average contact angles and corresponding standard derivation (SD).

Strain	Average ± SD
**Figure 1B**	
WT168	125.7° ± 6.58°
Δ*pspA*	130.4° ± 12.27°
Δ*samG*	130.2° ± 7.30°
Δ*samH*	131.5° ± 8.03°
Δ*samI*	0.0° ± 0.00°
Δ*pspA* Δ*samI*	5.7° ± 11.17°
Δ*samG* Δ*samI*	0.0° ± 0.00°
Δ*samH* Δ*samI*	116.8° ± 10.45°
Δ*samG* Δ*samH*	119.5° ± 5.00°
Δ*samG* Δ*samH* Δ*samI*	119.5° ± 5.27°
Δ*pspA-samGHI*	131.0° ± 9.28°

**Figure S1B**

WT168, LBGM	120.9° ± 10.24°
Δ*samI*, LBGM	0.0° ± 0.00°
WT168, MSgg	105.0° ± 11.23°
Δ*samI*, MSgg	0.0° ± 0.00°

**Figure S3B**	
WT168	123.4° ± 12.36°
Δ*samI*	0.0° ± 0.00°
NCIB 3610	130.5° ± 7.41°
NCIB 3610 Δ*samI*	134.6° ± 4.26°

**Figure S4A**	
WT168	127.8° ± 4.44°
Δ*samI*	0.0° ± 0.00°
Δ*samI* P_{hyper} *samH*	0.0° ± 0.00°
Δ*samI* P_{hyper} *samI*	0.0° ± 0.00°
Δ*samG* Δ*samI* P_{hyper} *samI*	29.7° ± 23.80°

**Figure S5**	
WT168, 0 % BnOH	135.4° ± 9.22°
WT168, 0.05 % BnOH	132.0° ± 5.89°
WT168, 0.1 % BnOH	134.6° ± 5.00°
WT168, 0.2 % BnOH	109.1° ± 19.31°
Δ*samI*, 0 % BnOH	0.0° ± 0.00°
Δ*samI*, 0.05 % BnOH	0.0° ± 0.00°
Δ*samI*, 0.1 % BnOH	0.0° ± 0.00°
Δ*samI*, 0.2 % BnOH	0.0° ± 0.00°
Δ*floA*, 0 % BnOH	134.9° ± 4.46°
Δ*floA*, 0.05 % BnOH	134.2° ± 6.73°
Δ*floA*, 0.1 % BnOH	132.8° ± 7.70°
Δ*floA*, 0.2 % BnOH	125.5° ± 16.24°
Δ*floT*, 0 % BnOH	125.5° ± 20.61°
Δ*floT*, 0.05 % BnOH	133.2° ± 4.41°
Δ*floT*, 0.1 % BnOH	135.1° ± 8.67°
Δ*floT*, 0.2 % BnOH	114.8° ± 19.03°
Δ*floAT*, 0 % BnOH	114.3° ± 10.85°
Δ*floAT*, 0.05 % BnOH	131.2° ± 4.52°
Δ*floAT*, 0.1 % BnOH	131.8° ± 6.02°
Δ*floAT*, 0.2 % BnOH	123.8° ± 13.05°

**Figure S8A**	
WT168	131.1° ± 6.44°
WT168, BslA-His	130.5° ± 3.42°
Δ*samI*	0.0° ± 0.00°
Δ*samI*, BslA-His	0.0° ± 0.00°

**Figure S10B**	
WT168	125.1° ± 10.16°
Δ*samI*	0.0° ± 0.00°
Δ*abrB*	123.2° ± 5.20°

ΔabrB ΔsamI	0.0° ± 0.00°
Δ*sinR*	133.2° ± 4.28°
Δ*sinR* Δ*samI*	128.4° ± 4.60°

3. References

Bach, J.N., and Bramkamp, M. (2013) Flotillins functionally organize the bacterial membrane. *Mol Microbiol* **88**: 1205-1217.

Donovan, C., and Bramkamp, M. (2009) Characterization and subcellular localization of a bacterial flotillin homologue. *Microbiology (Reading)* **155**: 1786-1799.

Edwards, D.H., Thomaides, H.B., and Errington, J. (2000) Promiscuous targeting of Bacillus subtilis cell division protein DivIVA to division sites in Escherichia coli and fission yeast. *EMBO J* **19**: 2719-2727.

Feddersen, H., Würthner, L., Frey, E., and Bramkamp, M. (2021) Dynamics of the Bacillus subtilis Min System. *mBio* **12**: e00296-00221.

Guttenplan, S.B., Shaw, S., and Kearns, D.B. (2013) The cell biology of peritrichous flagella in Bacillus subtilis. *Mol Microbiol* **87**: 211–229.

Koo, B., Kritikos, G., Farelli, J.D., Todor, H., Tong, K., Kimsey, H., Wapinski, I., Galardini, M., Cabal, A., Peters, J.M., Hachmann, A., Rudner, D.Z., Allen, K.N., Typas, A., and Gross, C.A. (2017) Construction and Analysis of Two Genome-Scale Deletion Libraries for *Bacillus subtilis*. *Cell Syst* **4**: 291–305.

Kovács, Á.T., and Kuipers, O.P. (2011) Rok regulates yuaB expression during architecturally complex colony development of *Bacillus subtilis* 168. *J Bacteriol* **193**: 998-1002.

Lewis, P.J., and Marston, A.L. (1999) GFP vectors for controlled expression and dual labelling of protein fusions in Bacillus subtilis. *Gene* **227**: 101-110.
